# Loss of AP-2delta reduces retinal ganglion cell numbers and axonal projections to the superior colliculus

**DOI:** 10.1186/s13041-016-0244-0

**Published:** 2016-06-04

**Authors:** Xiaodong Li, Frédéric Gaillard, Elizabeth A. Monckton, Darryl D. Glubrecht, Amit R. L. Persad, Markus Moser, Yves Sauvé, Roseline Godbout

**Affiliations:** Department of Oncology, Cross Cancer Institute, University of Alberta, 11560 University Avenue, Edmonton, AB T6G 1Z2 Canada; Department of Physiology, University of Alberta, 11560 University Avenue, Edmonton, AB Canada; Department of Ophthalmology, University of Alberta, 11560 University Avenue, Edmonton, AB Canada; Max-Planck-Institute of Biochemistry, Martinsried, Germany

**Keywords:** AP-2, Transcription factor, Retina, Ganglion cells, Axon, Brain, Superior colliculus, Electrophysiology

## Abstract

**Background:**

AP-2δ is the most divergent member of the Activating Protein-2 (TFAP2) family of transcription factors. AP-2δ is restricted to specific regions of the CNS, including a subset of ganglion cells in the retina. Retinal ganglion cells (RGCs), the only output neurons of the retina, are responsible for transmitting the visual signal to the brain.

**Results:**

*AP-2δ* knockout results in loss of *Brn3c* (*Pou4f3*) expression in AP-2δ -positive RGCs. While *AP-2δ-/-* mice have morphologically normal retinas at birth, there is a significant reduction in retinal ganglion cell numbers by P21, after eye opening. Chromatin immunoprecipitation indicates that *Brn3c* is a target of AP-2δ in the retina. Using fluorochrome-conjugated cholera toxin subunit B to trace ganglion cell axons from the eye to the major visual pathways in the brain, we found 87 % and 32 % decreases in ipsilateral and contralateral projections, respectively, to the superior colliculus in *AP-2δ-/-* mice. In agreement with anatomical data, visually evoked responses recorded from the brain confirmed that retinal outputs to the brain are compromised.

**Conclusions:**

AP-2δ is important for the maintenance of ganglion cell numbers in the retina. Loss of AP-2δ alters retinal axonal projections to visual centers of the brain, with ipsilaterial projections to the superior colliculus being the most dramatically affected. Our results have important implications for integration of the visual signal at the superior colliculus.

**Electronic supplementary material:**

The online version of this article (doi:10.1186/s13041-016-0244-0) contains supplementary material, which is available to authorized users.

## Background

AP-2s are a family of five transcription factors (AP-2α, β, γ, δ and ε) that function as homo- or heterodimers. AP-2α, AP-2β and AP-2γ are the most widely expressed and best-characterized of the five AP-2 transcription factors, with all three AP-2s implicated in neural crest formation [1–4]. In humans, AP-2α has been associated with orofacial defects and AP-2β with anxiety-related personality traits [5–7]. Knockout of *AP-2α, AP-2β, AP-2γ* and *AP-2ε* in mice indicates roles in craniofacial and limb development [8, 9], renal and adrenal chromaffin cell differentiation [10, 11], formation of extraembryonic lineages and primordial germ cell specification [12–14], and organization of the olfactory bulb [15], respectively. AP-2δ is the most divergent member of the AP-2 family [16] and is primarily found in heart as well as subsets of cells in the CNS [17, 18]. *AP-2δ−/−* mice are characterized by apoptosis in the inferior colliculus resulting in loss of this structure in adult mice [18]. Although the inferior colliculus is the main nucleus of the auditory pathway in midbrain, *AP-2δ−/−* mice still respond to sound, suggesting compensation through a different auditory route.

Three members of the AP-2 family (α, β and γ) are expressed in the amacrine and/or horizontal cells of the retina [19, 20]. We and others have previously reported that *AP-2δ* RNA is expressed in the ganglion cell layer of mouse and chick retina [21, 22]. Ectopic expression of AP-2δ in the developing chick retina results in extensive disruption of its layered structure, and the formation of large bundles of fibers that form perpendicular to the ganglion cell fiber layer, then run parallel to the ganglion fiber layer next to the retinal pigmented epithelium [23]. Putative AP-2δ target genes have been identified, including *Brn3c* and *Bhlhb4,* whose levels are significantly decreased in the midbrain of *AP-2δ−/−* mice [18, 24, 25].

*AP-2δ−/−* mice have not previously been examined for retinal or visual pathway defects. Here, we demonstrate the presence of AP-2δ in the same subset of retinal cells that express the retinal ganglion cell (RGC)-specific transcription factor Brn3c. While no gross disruption of retinal layers and ganglion fibers are observed upon *AP-2δ* knockout, both RGC numbers and RGC axonal projections to specific visual centers in the brain are altered in adult mice. In keeping with a role for AP-2δ in visual information processing, the post-photoreceptor synaptic response in the retina and the visually evoked response (VER) recorded from the visual cortex are impaired in *AP-2δ−/−* mice.

## Results

### AP-2δ is expressed in a subset of RGCs in wild-type mouse retina

**Fig. 1:**
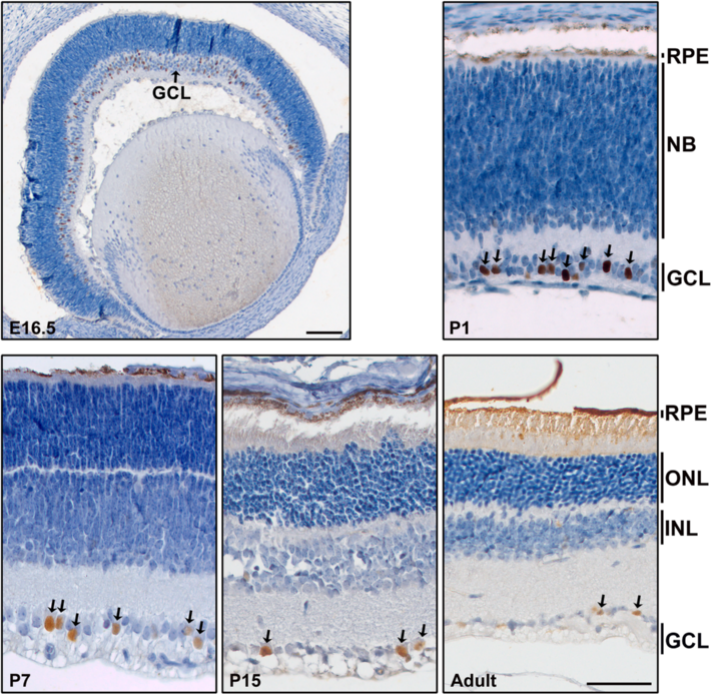
Immunohistochemical analysis of AP-2δ in mouse retina. Retinal tissue sections from E16.5, P1, P7, P15 and adult mice were immunostained with anti-AP-2δ antibody. The sections were counterstained with hematoxylin to label the nuclei. Photographs were taken with a 5× (E16.5) or 20× lens (P1, P7, P15 and adult) using a Zeiss Axioskop 2 plus microscope. The E16.5 image was assembled by taking overlapping photographs which were automatically merged in Photoshop. Scale bars = 100 μm for E16.5 tissue and 50 μm for P1, P7, P15 and adult tissues

**Fig. 2:**
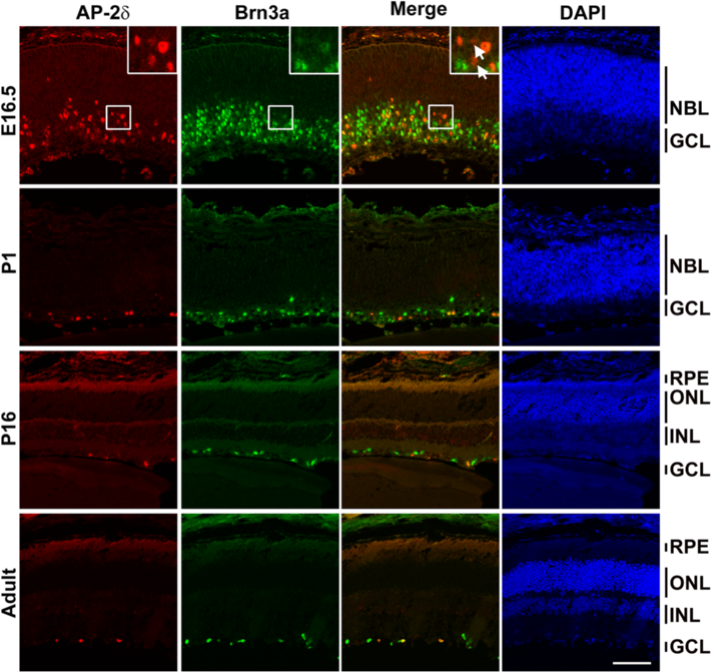
AP-2δ is expressed in a subset of RGCs in mouse retina. Retinal tissue sections from E16.5, P1, P16 and adult mice were co-immunostained with anti-AP-2δ and anti-Brn3a antibodies followed by secondary antibodies conjugated with Alexa 555 and Alexa 488, respectively. Sections were counterstained with DAPI to label the nuclei. The inset in E16.5 shows a magnified view of the area indicated by the square. Of the five AP-2δ-positive cells shown in the inset, two do not co-immunostain with Brn3a (indicated by *arrows*). Photographs were taken with a Zeiss LSM710 confocal microscope equipped with 20× lens. Abbreviations: NBL, neuroblastic layer; ONL, outer nuclear layer; INL, inner nuclear layer; GCL, ganglion cell layer. Scale bar = 100 μm

**Fig. 3:**
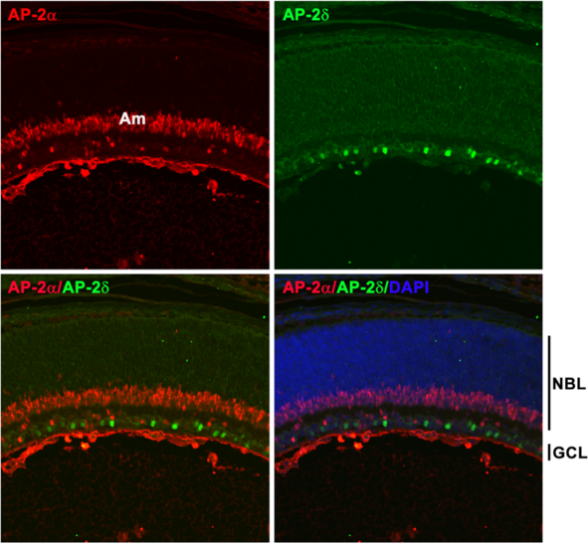
AP-2δ and AP-2α are found in different subsets of cells in the ganglion cell layer. Retinal tissue sections from wild-type mice at P1 were immunostained with anti-AP-2δ and anti-AP-2α (amacrine and displace amacrine marker) antibodies followed by secondary antibodies conjugated to Alexa 488 and Alexa 555, respectively. There was no overlap in cells expressing AP-2δ and AP-2α in the ganglion cell layer (see merged signal)

The temporal and spatial expression of AP-2δ in wild-type mouse retina was examined by immunohistochemistry. AP-2δ was detected in a subset of cells throughout the ganglion cell layer from embryonic day 16.5 (E16.5) through adulthood (Fig. 1). Labeling was also detected in a few cells in the inner nuclear layer, likely displaced RGCs [26]. To verify that AP-2δ-positive cells are indeed RGCs, we carried out co-immunostaining analysis of retinal sections using antibodies to AP-2δ and Brn3a, a well-established marker expressed in the majority of RGCs [26, 27]. AP-2δ co-localized with Brn3a-positive RGCs in the ganglion cell layer from E16.5 to adult, with all AP-2δ-positive cells co-immunostaining with Brn3a in P1 (125/125 cells, with counts compiled from 4 different tissue sections), P16 (158/158 cells – 8 different tissue sections) and adult retina (74/74 cells – 9 different tissue sections) (Fig. 2). Co-localization of Brn3a and AP-2δ was also observed in the inner nuclear layer, representing displaced ganglion cells [26, 28, 29]. At ED16.5, we observed a few AP-2δ-positive cells that appeared negative for Brn3a expression (~8-10/260 cells – 2 different tissue sections) (Fig. 2 – see inset). The absence of Brn3a in AP-2δ-positive cells at E16.5 may be due to delayed expression of Brn3a in these cells. To confirm that AP-2δ is only expressed in RGCs, we co-immunostained P1 retina with anti-AP-2δ and anti-AP-2α, a marker of amacrine and displaced amacrine cells [20, 21, 30]. There was no overlap in AP-2α and AP-2δ expression in the ganglion cell layer (Fig. 3).

Retinal maturation in wild-type mice was accompanied by a decrease in the number of AP-2δ-positive RGCs, from 33.7 % (423 AP-2δ+/1256 Brn3a + cells) and 30.8 % (74/240 cells) at E16.5 and P1, respectively, to 23.0 % (14/61 cells) in adult retina.

### *Brn3c* is a target of AP-2δ in the retina

**Fig. 4:**
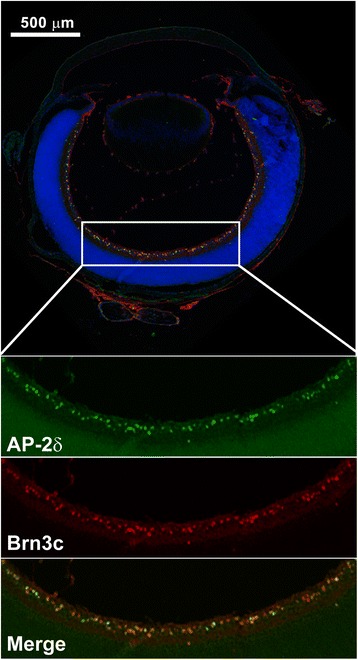
Co-expression of AP-2δ and Brn3c in retinal ganglion cells. P1 retinal tissue sections were immunostained with anti-AP-2δ and anti-Brn3c antibodies followed by secondary antibodies conjugated to Alexa 488 and Alexa 555, respectively. Sections were counterstained with DAPI to label the nuclei. Images were automatically assembled from multiple scans (4 × 4 scan; 4096 × 4096 pixels) using the Tile-scan function of the LSM program. The area outlined by the rectangle is magnified to show AP-2δ-positive cells (*green*), Brn3c-positive cells (*red*) and merged AP-2δ and Brn3c

**Fig. 5:**
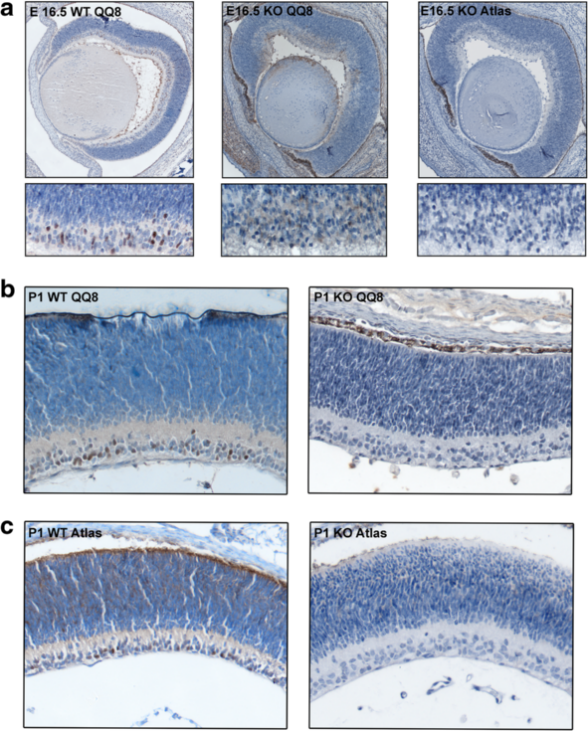
Loss of Brn3c expression in *AP-2δ−/−* retina. Paraffin-embedded tissue sections from E16.5 (**a**) and P1 (**b**, **c**) *AP-2δ+/+* and *AP-2δ−/−* retina were processed as previously described [81] and immunostained with two anti-Brn3c antibody: mouse anti-Brn3c (QQ8) (**a**, **b**) and rabbit anti-Brn3c antibody (Atlas) (**a**, **c**). The signal was detected using the Dako-Cytomation EnVision + anti-rabbit or anti-mouse secondary systems. Tissues were counterstained with the nuclear stain hematoxylin. Photographs were taken using a 20× lens. Images of the entire E16.5 retina sections were assembled from multiple overlapping photographs using the Adobe Photoshop merge function. The bottom panels in **a** show a higher magnification of the E16.5 ganglion cell layer

Previous work has shown that *Brn3c* is a likely target of AP-2δ in midbrain [18]. Like AP-2δ, Brn3c is expressed in a subset of RGCs [26, 31, 32]. We examined whether *Brn3c* might be a target of AP-2δ in mouse retina by co-immunostaining P1 retinal tissue sections with anti-AP-2δ and anti-Brn3c antibodies. Based on co-immunostaining data, there is close to 100 % overlap in AP-2δ-positive RGCs and Brn3c-positive cells (Fig. 4). To further investigate the possibility that the *Brn3c g*ene is a target of AP-2δ in the retina, we examined Brn3c distribution in the retinas of *AP-2δ+/+* versus *AP-2δ−/−* mice at E16.5, P1, P14 and adult. While Brn3c-expressing cells were observed in the ganglion cell layer of wild-type retina at all stages examined, there was a significant loss of Brn3c signal in the RGC layer of *AP-2δ−/−* mice, with many sections showing a complete absence of Brn3c expression (Additional file 1: Figure S1 – see E16.5 and P14) and some sections showing a few residual Brn3c-immunostained cells (Additional file 1: Figure S1 – see P1 and adult). To further investigate Brn3c expression in AP-2δ-deficient retina, we carried out immunohistochemical analysis with two different anti-Brn3c antibodies (Santa Cruz QQ8 and Sigma Atlas). While the QQ8 antibody occasionally resulted in non-specific staining, the Atlas Brn3c antibody showed complete absence of a Brn3c signal in *AP-2δ−/−* E16.6 and P1 retinas (Fig. 5 and Additional file 1: Figure S2). These results indicate that the residual Brn3c signal observed by immunofluorescence in *AP-2δ−/−* retina using the QQ8 anti-AP-2δ antibody is most likely non-specific. Loss of AP-2δ therefore results in loss of Brn3c expression in the retina.

**Fig. 6:**
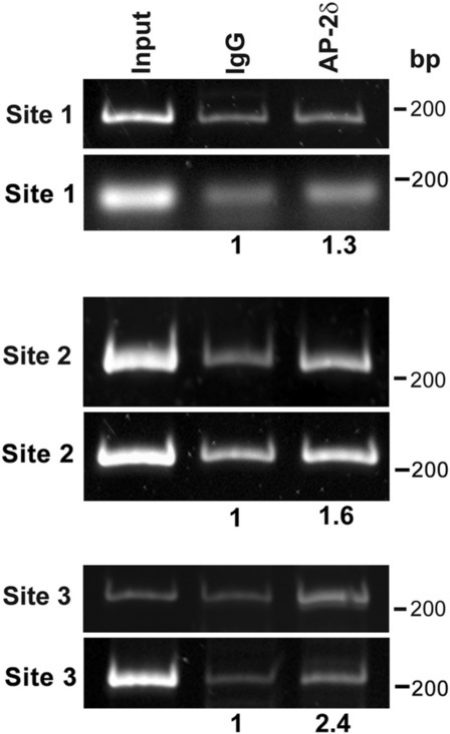
ChIP analysis of putative AP-2 binding sites located upstream of the *Brn3c* gene. P1 mouse retinal tissue was cross-linked and genomic DNA/AP-2δ complexes immunoprecipitated with anti-AP-2δ antibody. DNA purified from cross-linked complexes was PCR amplified using primer pairs flanking three putative AP-2 binding sites located within 3 kb of the *Brn3c* transcription start site. Normal rabbit IgG served as the negative control. Input DNA is the DNA following sonication but prior to immunoprecipitation. Results are shown from two experiments. Note that the second gel shown for site 1 was prepared using agarose rather than acrylamide. The intensity of the DNA signal was quantified by densitometric analysis using Adobe Photoshop and background subtraction. Values indicate the average of 2 independent experiments, with signal density in the IgG lanes set at 1

There are 3 putative AP-2 binding sites in the promoter region of *Brn3c*, one of which is a perfect AP-2 consensus binding site (site 2: GCCACAGGC, at −658 bp; conserved sequences are underlined). Sites 1 and 3 (GCCTCCCGGG at −180 bp and GCCTGAGGG at −2548 bp) are also well-documented AP-2 binding sites. Site 3 has previously been shown to be occupied by exogenous AP-2δ in Neuro2a cells [18]. Chromatin immunoprecipitation experiments were carried out using P1 mouse retina and anti-AP-2δ antibody. A >1.5× increase in band intensities was observed in the AP-2δ lanes compared to the IgG lanes for site 2 (average of 1.6×) and site 3 (average of 2.4×), suggesting that both these sites are occupied by endogenous AP-2δ in mouse retina (Fig. 6). Taken together, our data indicate that *Brn3c* is a direct target of AP-2δ in retina.

### Reduction in the number of RGCs in *AP-2δ*−/− mice

**Fig. 7:**
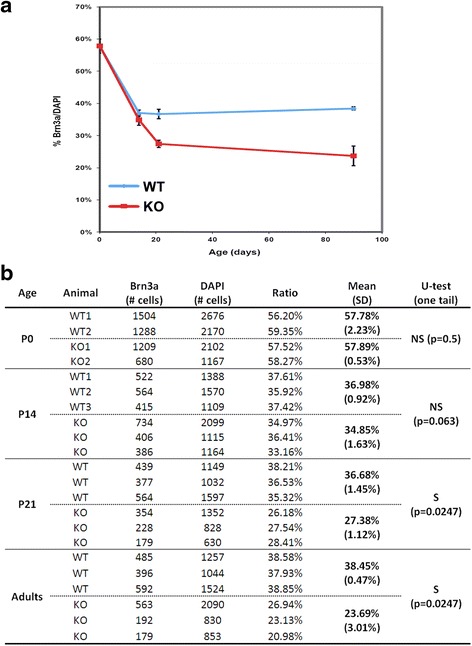
Decrease in RGC numbers in *AP-2δ−/−* mice. **a** Retinal tissue sections at P0, P14, P21 and adult stages were stained with anti-Brn3a antibody and DAPI. Graphical representation of the changes in the number of Brn3a-positive RGCs in wild-type versus *AP-2δ−/−* eyes at P0, P14, P21 and adult. **b** Brn3a-positive cells relative to DAPI-stained cells were counted throughout the ganglion cell layer of multiple sections from each of the eyes analysed. Significance of differences in the percentages of Brn3a-positive cells was calculated using the non-parametric Mann–Whitney U-test. Abbreviations: WT, wild-type; KO, knock-out; S, significant; NS, not-significant

Next, we examined whether AP-2δ knockdown affects RGC numbers. As RGCs only constitute ~50 % of cells in the ganglion cell layer [33], we immunostained P0, P14, P21 and adult *AP-2δ+/+* and *AP-2δ−/−* mouse retinal tissue sections with ganglion cell-specific anti-Brn3a antibody which recognizes the great majority (85.6 %) of RGCs [34] [Additional file 1: Figure S3 (P1 and P14) and Additional file 1: Figure S4 (P21 and adult)]. We then counted the number of Brn3a-positive relative to the total number of DAPI-stained cells in the ganglion cell layer (Fig. 7). There was no difference in the number of Brn3a-positive cells at P0 (57.8 % for *AP-2δ*+/+ compared to 57.9 % for *AP-2δ*−/−). Similarly, there was no significant change in RGC number at P14 (37 % for *AP-2δ*+/+ compared to 34.8 % for *AP-2δ*−/−). However, by P21, we observed a 25 % decrease in Brn3a-positive cells in *AP-2δ*−/− retina (36.7 % in wild-type retina compared to 27.4 % in *AP-2δ*−/− retina). The decrease in RGC numbers was more pronounced in adult *AP-2δ*−/− mice, with a 38 % decrease in the number of Brn3a-positive RGCs (38.4 % in wild-type retina compared to 23.7 % in *AP-2δ*−/−) (Fig. 7). Of note, the percentages of Brn3a-positive RGCs in the ganglion cell layer that we observed in wild-type retina are in keeping with previous reports [26, 34] and reflect the fact that by P14, approximately half of the cells in the ganglion cell layer are displaced amacrine cells. Thus, our results indicate that: (i) RGC numbers are significantly reduced in *AP-2δ−/−* mice and (ii) the majority of the reduction in RGC numbers occurs after eye opening at P14.

### RGC axons in *AP-2δ−/−* mice are able to take a correct path

RGCs send their axons through the optic disc to innervate the various visual centers of the brain. To investigate whether loss of AP-2δ expression in the retina affects axonal projections to the brain, we injected cholera toxin subunit B (CTB) conjugated to either Alexa 488 (green) or Alexa 594 (red) fluorescent dye into the vitreous chamber of *AP-2δ+/+* and *AP-2δ−/−* adult (3–4 month old) mice (Table 1). The experiment was terminated seven days after injection. Examination of flat-mounted retinas revealed no gross difference in the appearance and direction of optic fibers in wild-type versus *AP-2δ* knockout retina (Additional file 1: Figure S5). The thickness of the flat-mounted retinas and the strong signal intensity precluded quantitative analysis.

**Table 1:** Quantitative data for retinal axonal projections to the suprachiasmatic nucleus (SCN), dorsal lateral geniculate nucleus (dLGN), ventral lateral geniculate nucleus (vLGN), medial terminal nucleus (MTN) and superior colliculus (SC)

Structure	Measurement	W4-WT	AE1-WT	AF2-WT	Average	SD	T1-KO	W1-KO	AE2-KO	AF1-KO	Average	SD	KO vs WT; U-test statistics (2-tailed)
**SCN**	Volume contra proj (mm^3^)	0.0145	NA	0.0130	**0.0138**	**0.0011**	0.0114	0.0169	0.0119	0.0132	**0.0134**	**0.0025**	−2.9 %; NS (*P* = 0.64)
	Volume ipsi proj (mm^3^)	0.0129	NA	0.0125	**0.0127**	**0.0003**	0.0132	0.0119	0.0131	0.0116	**0.0125**	**0.0008**	−1.6 %; NS (*P* = 1)
**dLGN**	A-P length (max; μm)	940	910	1040	**963**	**68**	930	1022	1050	1130	**1033**	**83**	+7.3 %; NS (*P* = 0.29)
	Lat extension (μm)	900	780	720	**800**	**92**	750	780	720	750	**750**	**24**	−6.3 %; NS (*P* = 0.47)
	Volume contra proj (mm^3^)	0.1583	0.1284	0.1029	**0.1299**	**0.0277**	0.1146	0.1412	0.1389	0.1781	**0.1432**	**0.0262**	+10 %; NS (*P* = 0.48)
	Volume ipsi proj (mm^3^)	0.0207	0.0126	0.0161	**0.0165**	**0.0041**	0.0214	0.0168	0.0211	0.0139	**0.0183**	**0.0036**	+11 %; NS (*P* = 0.29)
	Ratio ipsi/contra	13.08 %	9.81 %	15.65 %	**12.85 %**	**2.92 %**	18.67 %	11.90 %	15.19 %	7.80 %	**13.39 %**	**4.64 %**	+4.2 %; NS (*P* = 0.16)
**vLGN**	A-P length (max; μm)	815	591	740	**715**	**114**	760	662	566	730	**680**	**86**	−4.9 %; NS (*P* = 0.48)
	Lat extension (μm)	450	360	450	**420**	**52**	360	450	450	510	**443**	**62**	+5.5 %; NS (*P* = 0.55)
	Volume contra proj (mm^3^)	0.0398	0.0407	0.0418	**0.0408**	**0.0010**	0.0325	0.0353	0.0383	0.0464	**0.0381**	**0.0060**	−7 %; NS (*P* = 0.29)
	Volume ipsi proj (mm^3^)	0.00699	0.00651	0.00668	**0.00673**	**0.00024**	0.00656	0.00496	0.00631	0.00449	**0.00558**	**0.00101**	−17 %; NS (*P* = 0.077)
	Ratio ipsi/contra	17.56 %	16.00 %	15.98 %	**16.51 %**	**0.91 %**	20.18 %	14.05 %	16.48 %	9.68 %	**15.10 %**	**4.41 %**	−8.8 %; NS (*P* = 0.72)
**MTN**	Volume contra proj (mm^3^)	0.00912	0.00755	0.00755	**0.00807**	**0.00091**	0.0112	0.0105	0.0096	0.00915	**0.01011**	**0.00092**	**+25.3 %; S (** ***P*** **= 0.034)**
	Volume ipsi proj (mm^3^)	none	none	none			none	none	none	none			
**SC**	A-P length (max; μm)	2100	1770	1930	**1933**	**165**	1765	1815	1680	1550	**1703**	**116**	−12 %; NS (*P* = 0.077)
	Lateral extension (μm)	1500	1530	1590	**1540**	**46**	1650	1620	1530	1560	**1590**	**55**	+3.2 %; NS (*P* = 0.21)
	Thickness contra proj (max; μm)	250	255	270	**258**	**10**	195	215	210	220	**210**	**11**	**−19 %; S (** ***P*** **= 0.032)**
	Volume contra proj (mm^3^)	0.526	0.454	0.504	**0.495**	**0.037**	0.315	0.414	0.302	0.321	**0.338**	**0.051**	**−32 %; S (** ***P*** **= 0.034)**
	Volume ipsi proj (mm^3^)	0.00654	0.00726	0.00817	**0.00732**	**0.00082**	0.00127	0.00104	0.00088	0.00051	**0.00093**	**0.00032**	**−87 % (~8 fold smaller); S (** ***P*** **= 0.034)**
	Ratio ipsi/contra	1.24 %	1.60 %	1.62 %	**1.49 %**	**0.21 %**	0.40 %	0.25 %	0.29 %	0.16 %	**0.28 %**	**0.10 %**	**−81 % (~5.3 fold smaller); S (** ***P*** **= 0.034)**

*Abbreviations*: *ipsi* ipsilateral, *contra* contralateral, *proj* projections, *S* significant; *NS* not significant, *KO* knockout, *WT* wild-type. Bold is used to indicate averages and standard deviations, as well as significant data in the last column

**Fig. 8:**
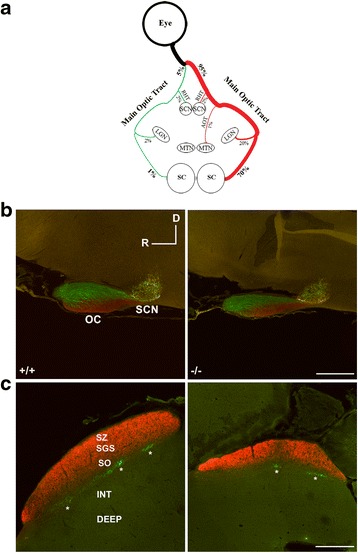
RGC projections to the optic chiasm (OC), suprachiasmatic nucleus (SCN) and superior colliculus (SC). **a** Diagram depicting the three major optic tracts [main optic tract, accessory optic tract (OAT) and retino-hypothalamic tract (RHT)] in brain and associated visual centers (nuclei) [SCN, lateral geniculate nuclei (LGN), medial terminal nucleus (MTN) and SC. **b, c** Sagittal brain sections from *AP-2δ+/+* and *AP-2δ−/−* mice intravitreally injected with CTB. Red, projections from the contralateral eye to the OC and SCN (**b**) and SC (**c**); green, projections from the ipsilateral eye to the OC and SCN (**b**) and SC (**c**). D, dorsal; R, rostral; SZ, stratum zonale; SGS, stratum griseum superficiale; SO, stratum opticum. Asterisks indicate clusters of ipsilateral projections. Scale bars = 200 μm (**b**) and 500 μm (**c**). Photographs were taken with a Zeiss LSM710 confocal microscope equipped with 10× lens

In mice, the great majority (95 %) of optic fibers cross to the opposite (contralateral) side of the brain at the optic chiasm (Fig. 8a) [35]. The gross morphology of the optic chiasm was similar in wild-type and *AP-2δ−/−* mice, with the ipsilateral tract always found dorsal to the contralateral tract (see OC in Fig. 8b). These combined data indicate that RGCs in *AP-2δ−/−* mice are able to: (i) form axons, (ii) reach the optic chiasm, and (iii) extend their axons towards subcortical visual targets.

While we did not perform CTB imaging on whole mount optic chiasm, we did measure the integrated optical density of both ipsilateral and contralateral projections at the very caudal pole of the optic chiasm using ImageJ software. These measurements were performed on 2 WT (AF2; W4) and 3 KO (AF1; AE2; T1) mice (4 sections per mouse). The optic chiasms of the remaining animals could not be measured because of poor image quality. The ipsilateral indexes (ipsilateral vs contralateral ratios) were an average of 1.378 ± 0.148 in WT (1.4678 for AF2; 1.2884 for W4) and an average of 1.064 ± 0.149 in KO (0.9569 for AF1; 1.2114 for AE2; 1.0230 for T1) mice. Thus, the ipsilateral index was decreased by 22.8 % in KO compare to WT, and this decrease was statistically significant (*p* = 0.0012; 2-tailed U-test). These data suggest a loss of ipsilateral projections in adult *AP-2δ−/−* mice and are consistent with the loss of RGCs observed in adult mice (Fig. 7).

### Projections to the suprachiasmatic nuclei (SCN) and lateral geniculate nuclei (LGN) are normal

After crossing the midline at the optic chiasm, optic fibers split into three major tracts: the retino-hypothalamic tract (RHT), the main optic tract and the accessory tract, each terminating at distinctive visual centers within the brain: suprachiasmatic nuclei (SCN) for the RHT, lateral geniculate nuclei (LGN) and superior colliculus (SC) for the main optic tract, and medial terminal nuclei (MTN) for the accessory optic tract (AOT) (Fig. 8a). Analysis of the axonal projections terminating at the SCN in wild-type and *AP-2δ−/−* mouse brains revealed similar volumes of ipsilateral and contralateral projections (see SCN in Fig. 8b; Table 1), with values in agreement with previous reports for wild-type mice [36–38]. Raw values indicate that terminal fields in *AP-2δ+/+* and *AP-2δ−/−* mice are equivalent. Thus, absence of AP-2δ does not affect projections along the RHT to the SCN.

The geniculate complex consists of three groups of nuclei: dorsal (d)LGN, ventral (v)LGN and intergeniculate leaflet. We first measured retinal projections to the dLGN. While there were small increases in the anterior-posterior length of the dLGN in *AP-2δ*−/− compared to *AP-2δ+/+* mice (+7.3 %), as well as in volumes of contralateral projections (+10 %) and ipsilateral projections (+11 %), these changes did not reach statistical significance (Table 1). Similarly, analysis of retinal projections to the vLGN magnocellular region (vLGN-mc, the lateral retinorecipient part of the LGN), revealed non-significant decreases in both the length and volume of retinal projections in *AP-2δ*−/− compared to *AP-2δ+/+* mice (Table 1). These results indicate that loss of AP-2δ does not affect the ipsilateral and contralateral fibers that travel along the main optic tract to the dorsal thalamus.

### *AP-2δ* knockout results in alterations in RGC axonal projections to the medial terminal nuclei (MTN) and superior colliculus (SC)

**Fig. 9:**
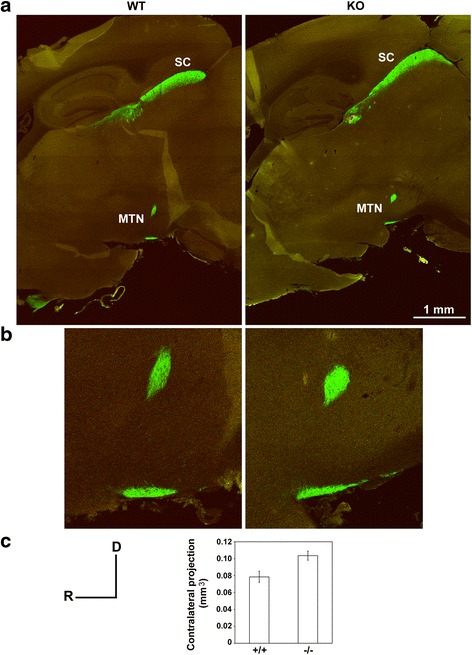
RGC projections to the medial terminal nuclei (MTN). **a**, **b** Sagittal brain sections (30 μm) from *AP-2δ+/+* (W4) and *AP-2δ−/−* (AF1) mice (intravitreally injected with CTB) were photographed with a 10× lens using the Tile-scan function of the LSM program. The image for the *AP-2+/+* mouse was constructed using a 12 × 10 tile scan, and the image for the *AP-2δ−/−* mouse was constructed using a 11 × 8 tile scan. Projections from the contralateral eye to the SC and MTN are shown in green, with a magnified view of the MTN shown in (**b**). **c** Graph depicting volume of contralateral projections to the MTN in three *AP-2δ+/+* and four *AP-2δ−/−* mice (values obtained from Table 1). Fiber volumes were measured in all sections containing components of the MTN. D, dorsal; R, rostral

The MTN is the major nucleus of the accessory optic tract. The volume of contralateral projections to the MTN was increased significantly (*p* = 0.034) by 25.3 % in *AP-2δ−/−* compared to *AP-2δ+/+* mice (Table 1) (Fig. 9). Ipsilateral projections to the MTN were not observed in either *AP-2δ+/+* or *AP-2δ−/−* mice. This result is likely related to the method used for tract tracing as a very weak ipsilateral input has previously been observed at the MTN using CTB immunochemistry [39], a technique that is more sensitive than the CTB-fluorescence technique used here.

The SC is a multi-layered structure that receives the bulk (at least 70 % in mouse) of axonal projections from the retina [40, 41]. The superficial layers of the SC (consisting of stratum zonale, stratum griseum superficiale and stratum opticum or optic layer) (Figs. 8c and 9a) receive direct retinal input and are sensory-related. The intermediate and deep layers of the SC receive input from both sensory (including the superficial layers of the SC) and motor structures. The retinal projections to the SC are bilateral, but the contralateral projections are much denser.

The superficial layers of the SC were evenly labeled by contralateral projections, with these projections identified in a similar number of brain sections in *AP-2δ+/+* (53 ± 3) and *AP-2δ−/−* (50 ± 3) mice. However, both the thickness and the volume of contralateral projections to the SC were significantly reduced, by 19 % (*p* = 0.032) and 32 % (*p* = 0.034), respectively, in *AP-2δ*−/− mice (Table 1). The most striking effect of *AP-2δ* knockout was in the number of terminal clusters and volume of ipsilateral (labeled in green in Fig. 8c) projections to the SC. An 87 % reduction in the volume of ipsilateral projections was observed in *AP-2δ*−/− mice (Fig. 8c; Table 1). Ipsilateral clusters in the optic layer of the SC were reduced to *n* = 1 (rarely 2) per section in *AP-2δ−/−* mice compared to *n* = 2–5 per section in wild-type mice. Individual ipsilateral clusters are indicated by an asterisk in Fig. 8c, with three clusters shown in *AP-2δ+/+* mice and 2 clusters shown in *AP-2δ−/−* mice. Moreover, whereas ipsilateral projections were identified in 40 ± 2 successive brain sections in wild-type mice, they were found in only 18 ± 4 successive brain sections in *AP-2δ−/−* mice. Thus, there is considerably reduced input of retinal projections, especially ipsilateral projections, to the SC in adult *AP-2δ−/−* mice.

Next, we examined whether reduced retinal projections to the SC might be explained by a reduction in the size of the SC. Although AP-2δ knockout was accompanied by a 12 % decrease in the maximum posterior-anterior length of the SC (Table 1), a 3.2 % increase in the lateral extension of the SC (Table 1) and a 9 % decrease in the full extension of the contralateral projections at the very dorsal surface of the SC (2387 ± 133 μm in *AP-2δ+/+* vs 2171 ± 143 μm in *AP-2δ−/−* mice), none of these variations were statistically significant. Furthermore, measurements for knockout animals showed little deviation from the lower limit of values reported in the literature for wild-type mice with normal SC (maximum A-P length of 1750–2000 μm; lateral extension of 1490–1550 μm; dorsal extension of 2200–2300 μm) [42, 43] (our own data on C57BL/6 mice).

### Visual electrophysiology

**Fig. 10:**
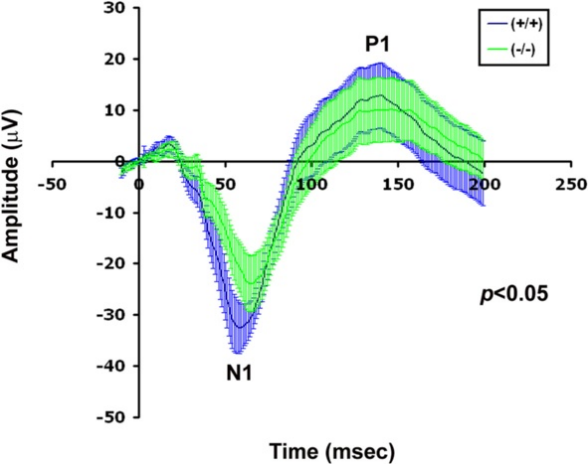
Visually evoked response in *AP-2δ+/+* and *AP-2δ−/−* mice. VER traces in *AP-2δ+/+* and *AP-2δ−/−* mice. Response amplitude (μV, *y-axis*) is shown as a function of time (*x-axis*). Traces of both *AP-2δ+/+* mice (*blue*) and *AP-2δ−/−* mice (*green*) are shown as filled-in lines which include the maximum and minimum amplitudes recorded for each mice at the indicated times. The solid lines represent amplitude averages for *AP-2δ+/+* and *AP-2δ−/−* mice

We examined the effect of *AP-2δ−/−* knockdown on the visual cortical responsiveness by recording full field VER. Superimposed averaged traces from wild-type and *AP-2δ−/−* mice are presented in Fig. 10. VER amplitudes for N1 (negative component) and P1 (positive component) were reduced in *AP-2δ−/−* mice (24 ± 5 and 34 ± 5 μV for N1 and P1) compared to wild-type littermates (33 ± 5 and 46 ± 3 μV for N1 and P1). In addition, N1 latencies were delayed in *AP-2δ−/−* mice (65 ± 3 msec) compared to wild-type littermates (58 ± 3 msec). There was no difference in P1 latencies in *AP-2δ−/−* mice (146 ± 5 msec) compared to wild-type littermates (140 ± 5 msec). These alterations in the VER waveform are in agreement with impairment in the functional integrity of the retino-geniculo-cortical system in *AP-2δ*−/− mice.

**Fig. 11:**
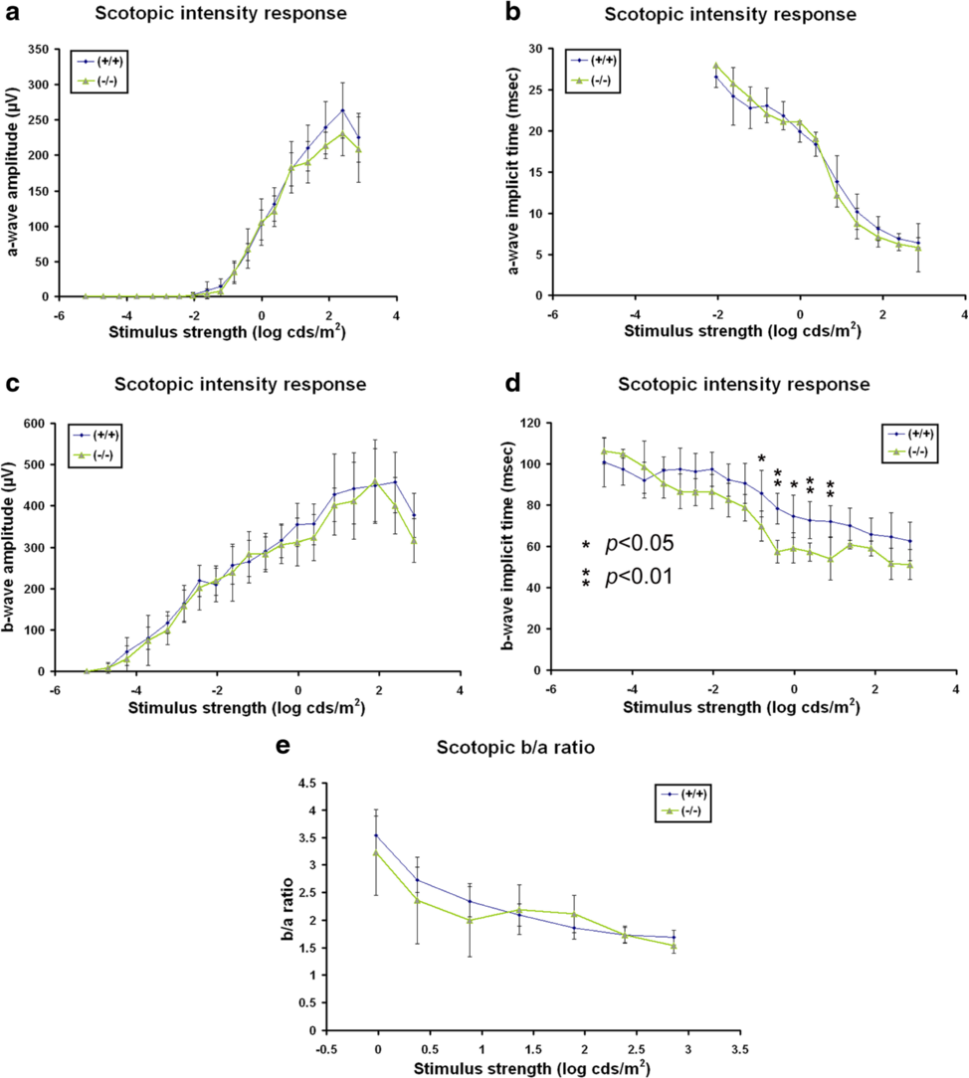
Dark-adapted intensity responses in *AP-2δ+/+* and *AP-2δ−/−* mice. Recordings of dark-adapted ERG a-waves (**a**, **b**) and b-waves (**c**, **d**) in *AP-2δ+/+* and *AP-2δ−/−* mice elicited by step-wise increases in stimulus strength. ERG a-wave and b-wave amplitudes as a function of stimulus strength are shown in **a** and **c**. ERG a-wave and b-wave implicit times as a function of stimulus strength are shown in (**b** and **d**). **e** Dark-adapted b-wave to a-wave ratio

**Fig. 12:**
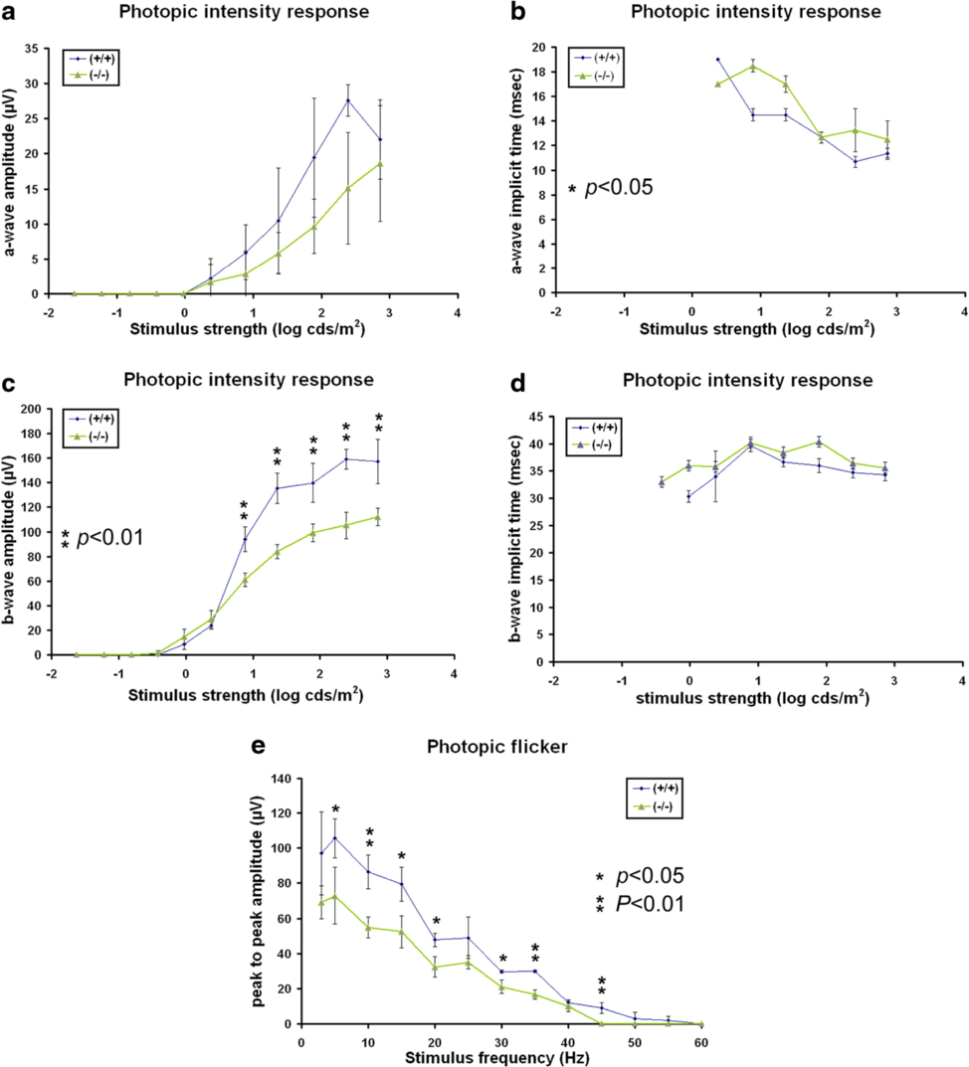
Photopic intensity responses in *AP-2δ+/+* and *AP-2δ−/−* mice. Recordings of lighted-adapted ERG a-waves (**a**, **b**) and b-waves (**c**, **d**) in *AP-2δ+/+* and *AP-2δ−/−* mice elicited by step-wise increases in stimulus strength. ERG a-wave and b-wave amplitudes as a function of stimulus strength are shown in (**a** and **c**). ERG a-wave and b-wave implicit times as a function of stimulus strength are shown in (**b** and **d**). **e** ERG recordings of light-adapted flicker responses as a function of stimulus frequency

To address a potential retinal contribution to the abnormal VER in *AP-2δ−/−* mice, we used full field electroretinography (ERG) to assess retinal function. Measurements were made under both scotopic (dim light) and photopic (bright light) conditions. The combined scotopic and photopic ERG data indicate that *AP-2δ−/−* mice retain normal rod and cone photoreceptor responses (as indicated by the a-waves). However, there was a specific reduction in mixed b-wave amplitudes, reflecting defective post-synaptic processing (Figs. 11 and 12). Preservation of pure-b-wave amplitudes (elicited by stimuli sub-threshold to cones, <2.0 log cds/m^2^) combined with reduced photopic flicker amplitudes suggest that this defect is post-synaptic to cones.

### Expression of AP-2δ in superior colliculus

**Fig. 13:**
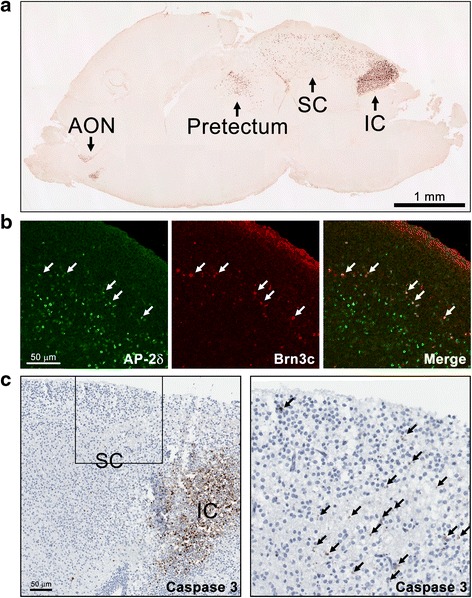
AP-2δ expression and apoptosis in the superior colliculus. (**a**) Wild-type P1 mouse brain sagittal section immunostained with anti-AP-2δ antibody. Photographs were taken with a 5× lens and photo-merged in Photoshop. A positive signal is observed in the inferior colliculus (IC), superior colliculus (SC), pretectum and anterior olfactory nucleus (AON). **b** Sagittal section from wild-type P1 brain co-immunostained with anti-AP-2δ and anti-Brn3c antibodies followed by secondary antibodies conjugated with Alexa 488 and Alexa 555, respectively. AP-2δ-positive cells are observed throughout the SC, with Brn3c cells co-expressed with AP-2δ (e.g. see *arrows*) in a subset of AP-2δ-positive cells. **c** Sagittal section from *AP-2δ−/−* P1 brain immunostained with anti-caspase 3 antibody showing apoptosis in the inferior colliculus and to a much lesser extent in the SC. Inset is magnified on the right. *Arrows* point to apoptotic cells

Sagittal sections of wild-type P1 mouse brain immunostained with anti-AP-2δ antibody revealed the expected strong AP-2δ signal in the inferior colliculus (Fig. 13a) [18]. AP-2δ-positive cells were also observed in the anterior olfactory nucleus (AON), the pretectum (anterior pretectal nucleus) and a subset of cells throughout most of the SC. Next, we co-immunostained wild-type P1 mouse brain tissue with anti-AP-2δ and anti-Brn3c antibodies. A subset of AP-2δ-positive cells in SC expressed Brn3c (Fig. 13b), with most Brn3c-positive cells found immediately below the optic layers. Strikingly, another member of the AP-2 family virtually absent in the inferior colliculus, AP-2β, was expressed throughout most of the SC (Additional file 1: Figure S6).

**Fig. 14:**
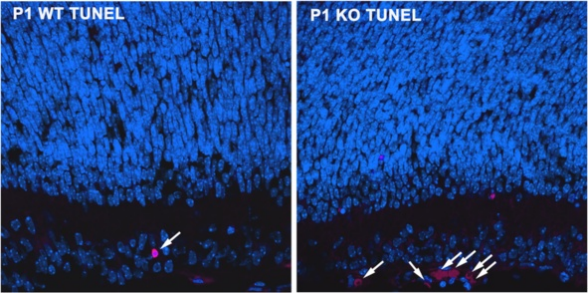
Increased apoptosis in the ganglion cell layer of *AP-2δ−/−* P1 retina. Apoptotic cells in P1 retinal tissue sections from *AP-2δ+/+* (WT) and *AP-2δ−/−* (KO) mice were stained using the In Situ Cell Death Detection kit, TMR red (Roche). Photographs were taken using a Zeiss LSM510 confocal microscope. *Arrows* point to apoptotic cells

The inferior colliculus of *AP-2δ−/−* mice undergoes apoptosis during development [18]. To investigate whether apoptosis might also be occurring in the SC, we examined the expression of cleaved caspase 3 in *AP-2δ−/−* P1 mouse brain. Apoptotic cells were detected in both the superficial and deep layers of the SC; however, the number of apoptotic cells was much lower than that observed in the inferior colliculus (Fig. 13c), suggesting a more limited or gradual loss of cells in the SC. Of note, the number of apoptotic cells in the ganglion cell layer of P1 retina was also elevated in *AP-2δ−/−* versus *AP-2δ+/+* mice (Fig. 14).

## Discussion

The highly restricted expression of AP-2δ in subsets of CNS neurons suggests specialized roles for this transcription factor. In mice, AP-2δ is highly expressed in the inferior colliculus, a major auditory center located in the posterior dorsal midbrain. AP-2δ is the only member of the AP-2 family expressed in this structure [18]. Accordingly, the most obvious phenotype observed in *AP-2δ−/−* mice is loss of the inferior colliculus through apoptosis [18]. As AP-2δ is expressed in a subset of RGCs in the retina, we investigated whether AP-2δ might also play a role in vision. Here, we show that loss of AP-2δ affects the visual pathway in adult mice, as demonstrated by: (i) a reduced number of RGCs in the retina, (ii) marked reduction in the volume of retinal projections to the SC (especially those arising from the ipsilateral retina), and (iii) post-photoreceptor deficiencies in response to light.

### Retinal defects in *AP-2δ−/−* mice

Brn3’s are a family of transcription factors expressed in overlapping patterns in RGCs during mouse development: Brn3b is expressed at E11.5 [44], Brn3a at E12.5 [45] and Brn3c at E14.5 [31]. Knockout of *Brn3b* results in loss of ~75 % of RGCs, with the remaining RGCs showing axonal defects [27, 46, 47]. Conditional knockout of *Brn3a* in the retina affects RGC dendritic morphology and stratification [27]. Although there is no apparent retinal defect in *Brn3c−/−* mice, knockout of both *Brn3b* and *Brn3c* results in decreased RGC survival and axonal outgrowth compared to *Brn3b* knockout alone [31]. Moreover, whereas *Brn3b−/−* mice have an ipsilateral to contralateral axonal ratio of ~1 at the optic chiasm, *Brn3b/Brn3c* double knockouts have virtually no ipsilateral projections, indicating a role for Brn3c in specification of ipsilateral axonal projections [31]. Of note, only 3 of 15 morphologically-defined RGC subtypes express Brn3c, suggesting a specialized role for Brn3c in a subset of RGCs [32]. *Brn3c* has previously been postulated to be a target of AP-2δ in midbrain [18]. Our ChIP data support *Brn3c* being a target of AP-2δ in retina. Accordingly, AP-2δ-positive cells in wild-type retina express Brn3c and loss of AP-2δ is accompanied by loss of Brn3c expression in the retina.

Developmentally-regulated cell death in wild-type RGCs peaks at P2-P5 and ends at ~ P11 [48]. There was no significant difference in the numbers of Brn3a-positive cells in *AP-2δ+/+* versus *AP-2δ−/−* retinas at P1 and P14, although an increased number of apoptotic cells was observed in the retina of *AP-2δ−/−* mice at P1. However, there was a decrease in Brn3a-positive RGC numbers at P21 in *AP-2δ−/−* retina, culminating in a 38 % loss in adult mice. These results suggest early stage compensation for loss of RGCs and/or a gradual loss of RGC cells starting at P1. Based on our data, 30.8 % (at P1) to 23 % (in adults) of RGCs co-express Brn3a and AP-2δ in wild-type mice. Thus, more RGCs are lost than can be accounted for by loss of all RGCs of the *AP-2δ* lineage in *AP-2δ−/−* mice. We offer two possible explanations for this discrepancy. First, there may be prolonged degeneration of RGCs, starting from those cells in which AP-2δ would normally be expressed, but extending to RGCs that don’t normally express AP-2δ, due to accumulation of toxic debris, decreased neurotrophic support and/or neurotransmitter blockade in the ganglion cell layer of *AP-2δ−/−* mice [49]. In this regard, it is noteworthy that *Bdnf*, a putative target of Brn3c, is downregulated in *AP-2δ−/−* mice [18, 50]. *Bdnf* encodes a neurotrophin growth factor shown to rescue RGCs from neurotoxic death [51]. Second, higher than predicted RGC death may be caused by loss of targets for RGC axons in the SC of *AP-2δ−/−* mice as described by other groups [52–55]. For example, Carpenter *et al.* observed substantial reduction in RGC numbers upon injury to the SC in the first 4 post-natal days (P4), but not past P7 [52]. As shown here, apoptotic cells are detected in the SC of *AP-2δ−/−* mice as early as P1, which may in turn affect the long term survival of a subset of RGCs projecting to the SC. In keeping with the idea that loss of expression of AP-2δ in the SC during brain development may affect RGC numbers, Perry and Cowey showed that SC ablation leads to RGC degeneration and aberrant projections in 1-day old rats, albeit not in adult rats [56, 57].

### Axonal projection defects in *AP-2δ−/−* mice

In mice, 95 % of RGC axons cross to the opposite (contralateral) side at the optic chiasm. By injecting fluorescent-tag CTB in the eyes of *AP-2δ−/−* mice, we showed that RGC axons display grossly normal intraretinal fasciculation, with both contralateral and ipsilateral axonal projections observed at the optic chiasm. However, compared to wild-type mice, the ipsilateral index in *AP-2δ−/−* mice was reduced by 22.8 %. As the number of Brn3a-positive RGCs is reduced by 38 % in adult *AP-2δ−/−* compared to *AP-2δ+/+* mice, these results indicate that in addition to the ipsilateral pathway, a significant fraction of the dying RGCs must also belong to the contralateral pathway. In support of both contralateral and ipsilateral retinal projections being affected by loss of AP-2δ, ipsilateral and contralateral projections are both reduced in the SC of *AP-2δ−/−* mice (see below) and the contralateral projection field appears to be entirely missing in the dorsal roof of the anterior pretectal nucleus (data not shown) [39]. As a point of interest, ipsilateral fibers would be absent at the chiasma level in adult *Brn3b/Brn3c* knockout mice [31].

No significant differences in contralateral and ipsilateral projections to the SCN were observed between wild-type littermates and *AP-2δ−/−* mice. These results are consistent with previous reports indicating that Brn3c-positive RGCs do not project to the SCN in mice [27, 58]. We also found no differences between *AP-2δ+/+* and *AP-2δ−/−* mice in contralateral and ipsilateral projections to the LGN. Retinal input to the dLGN is established from E16 to P8 [59] and involves Brn3a-, Brn3b- and Brn3c-expressing RGCs [27, 58]. As there is no loss in RGC numbers in *AP-2δ−/−* mice up to P14, it is possible that the dLGN receives input from a normal number of RGCs during its establishment, and that expression of Brn3a and Brn3b in these RGCs can compensate for loss of Brn3c.

While not a major target for axonal projections, the MTN does relay information for the control of vertical optokinetic nystagmus and receives input from ON, direction-selective RGCs [60]. Compared to wild-type mice, a significant increase (25 %) in contralateral projections to the MTN was observed in adult *AP-2δ−/−* mice. While unexpected, it is unlikely that this observation is due to technical bias as similar results were obtained whether thresholded images were used or raw RGB data (Additional file 1: Table S1). Retinal input to the MTN is dependent on Brn3a- and Brn3b-expressing RGCs, but not Brn3c-expressing RGCs [27, 58]. As Brn3c is expressed in a subset of Brn3a and Brn3b-expressing RGCs [26], *AP-2δ−/−* RGCs will still express Brn3a and Brn3b. It is possible that axons from some of these Brn3a+/Brn3b+/Brn3c- RGCs are rerouted to the accessory optic tract during development, resulting in an increased volume of contralateral projections in the MTN*.* Furthermore, RGC projections to visual centers have been shown to redistribute up to the time when they reach their natural termination sites [56, 57]. Retinal afferents in neonatal rats have been found to extend beyond the MTN proper, sending numerous collateral branches into the adjacent ventral tegmental area before gradually retracting to form the adult projection pattern observed in P12-16 [61].

The SC is a major target for retinal fibers in mammals, with at least 70 % of RGCs projecting to this primary visual center in mice [40, 41]. The bulk of fibers to the SC are established between E16.5 and P1/P2, with most of the projections (~98 %) coming from the contralateral side [62–64]. Retinal projections are initially diffuse, with aberrant branches and axons eliminated between P3 and P8. The final map is established at P12 just before the eyes open at P14 [62]. Similar to the dLGN, the SC receives input from Brn3a-, Brn3b- and Brn3c-positive fibers [27]. However, in contrast to the dLGN, we observed a 32 % decrease in the volume of contralateral projections and an 87 % decrease in the volume of ipsilateral projections to the SC in *AP-2δ−/−* mice. This massive decrease in retinal projections to the SC in *AP-2δ−/−* mice is consistent with: (i) the large population of RGCs projecting to the SC, and (ii) the death of a significant proportion of RGCs (and possibly SC cells) in *AP-2δ−/−* mice post-P14. The SC (along with the dorsal roof of the anterior pretectal nucleus; Fig. 13a) are the only brain structures known to have AP-2δ/Brn3c-expressing cells within their retinorecipient domains. The loss of retinal input to these structures could therefore be explained by loss of AP-2δ expression in both RGCs and brain structures receiving retinal input.

The decrease in the ipsilateral index at the chiasma level and the loss of most ipsilateral terminal field clusters observed in *AP-2δ−/−* mice suggest that AP-2δ plays a major role in controlling the production/pathfinding of ipsilateral retinal projections to the SC. In keeping with this observation, *Brn3c* (an AP-2δ target gene) promotes the guidance of ipsilateral retinal projections [31]. While an 87 % reduction in ipsilateral projections to the SC sounds impressive, it must be remembered that these projections are derived from ~2 % of the RGC population, suggesting that loss of AP-2δ primarily affects RGCs projecting contralaterally to the SC. Nevertheless, the extensive loss of ipsilateral projections to the SC suggests that there may be preferential loss of RGCs in the temporal-ventral crescent, the location of the RGCs that send ipsilateral projections to the SC [42, 65, 66].

A primary role for the SC is the generation and control of eye movement or gaze shifts involving both the eye and head [40]. As such, the SC serves as a focus for a multitude of visual-sensory integrations and goal-oriented visual/sensory-motor responses required for tracking targets. Ipsilateral projections to the SC are required for binocular vision [67]. The decrease in projections to the SC observed in *AP-2δ−/−* mice may therefore affect a variety of optical and multisensory integrative processes. Future work will involve examining the behavior of *AP-2δ−/−* mice, especially as related to vision or vision-motor integration.

The inferior colliculus located immediately next to the SC in the midbrain, is absent in adult *AP-2δ−/−* mice [18]. The inferior colliculus provides extensive auditory input to the deep layers of the SC [68]. In turn, anatomical tracings have shown that the inferior colliculus receives visual input from the SC, as well as from retina and visual cortex [69–72], although a recent study in mouse indicates that retinal projections to the inferior colliculus are sparse [39]. Furthermore, electrophysiological analyses of the anesthesized ferret suggest that the source of the visual responses in the inferior colliculus is the SC rather than direct input from the retina or cortex [73]. Therefore, it is unlikely that absence of the inferior colliculus would directly affect RGC numbers and axonal projections to the SC in *AP-2δ−/−* mice; however, it is highly likely that absence of the inferior colliculus can affect SC response, especially as related to visual and auditory signal integration in midbrain.

### Functional defects in *AP-2δ−/−* mice

We used full field ERG to assess the functional integrity of the retina in adult *AP-2δ−/−* mice. The observed normal a-wave amplitudes and implicit times under both scotopic and photopic conditions indicate that the function of rod and cone photoreceptors themselves is not affected by loss of AP-2δ expression. Furthermore, our finding of normal pure rod scotopic b-waves [74] in *AP-2δ−/−* mice is in agreement with an intact rod-bipolar cell pathway. The observed reductions in the amplitudes of the photopic b-wave and flicker response (both reflecting pure cone-driven post-synaptic activity) in *AP-2δ−/−* mice are characteristic of defective bipolar cells, while the functional integrity of cones themselves is spared. We also observed an increase in the b-wave implicit time under dark adaptation at stimulus strengths exceeding −1 log cds/m^2^, which falls in the sensitivity range of both rods and cones (referred to as a mixed b-wave). Therefore, cone-driven contribution alone to these mixes b-waves could account for the amplitude reduction observed in *AP-2δ−/−* mice.

Previous studies on the importance of RGCs to the full field ERG indicate that RGCs contribute to b-waves generated by rod (rather than cone) bipolar cells [75, 76]. Furthermore, reductions in rod-driven b-wave amplitudes were observed following an estimated loss of 90 % of all RGCs two weeks after intraorbital transection of the optic nerve [75, 76]. Thus, it is unlikely that the 38 % reduction in RGCs observed in *AP-2δ−/−* mice can account for our findings of reduced cone-driven b-wave and flicker amplitudes. We speculate that the effects on full field ERG observed in *AP-2δ−/−* mice are caused by defects in retinal cell types other than RGCs (e.g. cone bipolar cells).

The VER is dependent on the functional integrity of the retinocortical pathway, with the dLGN being responsible for transmitting retinal input to the cortex. When recorded under light-adapted conditions, the VER is a measure of cone-driven pathway activity [77]. Any defect in this pathway will alter the VER waveform. It is therefore possible that reduced amplitudes (~27 %) and prolonged latencies of the VER observed in *AP-2δ−/−* mice are caused by compromised retinal output to the dLGN even though the differences observed in RGC projections to the dLGN were not significantly different from that of wild-type mice. However, as: (i) the SC sends projections to the lateroposterior thalamic nucleus as well as to the dLGN [40, 78], and (ii) indirect tectocortical projections have been documented in rats [79, 80], it remains possible that the abnormal VER responses in *AP-2δ−/−* mice are caused by defective retinotectal projections, in keeping with our anatomical results. Together, our electrophysiological findings are in agreement with decreased RGC numbers observed in *AP-2δ−/−* mice and suggest defects in post-photoreceptor processing of cone-driven visual information.

## Conclusions

Our data demonstrate that loss of AP-2δ function during development can have phenotypically profound consequences (loss of the inferior colliculus) as well as more subtle consequences (reduced numbers of RGCs and RGC axons projecting to the SC, as shown in this study). We show that AP-2δ is important for maintenance of RGC numbers as well as the fine-tuning of axonal connections to visual centers in the brain. We propose that absence of AP-2δ promotes redistribution of RGC projections, resulting in increased projections to the MTN and decreased projections to the SC. The latter, in turn, may enhance RGC degeneration through a SC-retina feedback mechanism as described in the section on retinal defects. Our results suggest that projections of axons from AP-2δ-positive RGCs to the SC may play an important role in the integration of the visual signal with other sensory and motor signals.

## Methods

### Animals

The generation of the *AP-2δ-*deficient mouse line has been previously described [18]. The mice were maintained in the *Mus musculus* C57BL/6 background by heterozygous crosses. Every 3 or 4 generations, heterozygous mice were mated to C57BL/6 mice purchased from Charles River to prevent genetic drift. All animal studies were conducted in accordance with the Canadian Council on Animal Care (CCAC) Guidelines and Policies and in compliance with the ARRIVE guidelines with approval from the Cross Cancer Institute Animal Care Committee.

### Immunohistochemistry and immunofluorescence analysis

Mouse eyes and brains were either (i) fixed in formalin and embedded in paraffin, or (ii) fixed in 4 % paraformaldehyde, cryoprotected in sucrose and embedded in OCT, and then processed as previously described [81]. The primary antibodies used include: rabbit anti-AP-2δ#1 (for ChIP analysis) (1:1000 dilution) [22], rabbit anti-AP-2δ#2 (for immunostaining) (1:2500) [18], mouse anti-Brn3a (Cat. # MAB1585; 1:200, Chemicon), mouse anti-Brn3c (QQ8 Cat. # sc-81980; 1:100, Santa Cruz Biotechnology), rabbit anti-Brn3c (Atlas Cat. # AV33065; 1:800, Sigma), rabbit anti-cleaved caspase 3 (Cat. # AB3623; 1:500, Chemicon), rabbit anti-AP-2β (Cat. # 2509; 1:200, Cell Signaling Technology) and mouse anti-AP-2α (3B5, 1:250; developed by Dr. Trevor Williams and obtained from the Developmental Studies Hybridoma Bank developed under the auspices of the NICHD and maintained by the University of Iowa). Apoptotic cells in retina were detected using the In Situ Cell Death Detection kit, TMR red (Roche) which is based on labeling of DNA strand breaks that occur at early stages of apoptosis (TUNEL assay).

RGCs in wild-type and *AP-2δ−/−* littermates were counted using 8-μm tissue sections from different regions of the retina. Three to four tissue sections were counted for each mouse analyzed at P0, P14, P21 and adult. Tissue sections were immunostained with mouse anti-Brn3a antibody in order to identify RGCs in the ganglion cell layer. The DNA was stained with DAPI (nuclear stain) to identify all the cells in the ganglion cell layer. Percentages of RGCs were determined by dividing the total number of Brn3a-positive cells found throughout the ganglion cell layer with the total number of DAPI-stained cells in this layer. Cells were counted throughout the retina with the exception of folded areas. Brn3a-positive cells and DAPI-stained cells in each tissue section were counted by two independent observers and averaged. Counts by the two observers were generally within 5 % of each other.

### Endogenous chromatin immunoprecipitation

P1 mouse retinal tissue from 5 wild-type pups was dissociated with trypsin and cross-linked with 1 % formaldehyde for 10 min at room temperature. Cells were homogenized in lysis buffer (44 mM Tris–HCl pH 8.0, 1 % SDS, 10 mM EDTA, 1 mM PMSF and Roche Complete protease inhibitors) and sonicated for 30 cycles with pulses of 30 s on/30 s off (Bioruptor 300, Diagenode). After sonication, the lysates were pre-cleared by incubation with protein A-Sepharose beads. The precleared lysates were immunoprecipitated with affinity-purified anti-AP-2δ antibody [22] or rabbit IgG (negative control). Protein-DNA complexes were eluted from the beads. Crosslinks were reversed, protein digested with proteinase K, and the DNA purified with the QIAquick PCR purification kit. PCR was carried out using primers flanking three putative AP-2 binding sites located upstream of the *Brn3c* transcription initiation site. Site 1 (GCCTCCCGGG, at −180 bp relative to the transcription start site as defined by accession number NC_000084) was amplified using forward primer 5′-CAGGCCGGGGTATAAATGC-3′ and reverse primer 5′-TCTCCCCCACCTGCTTCTT-3′; site 2 (GCCACAGGC, at −658 bp) was amplified using forward primer 5′-GCCCACAAGTTCTGTTTCTC-3′ and reverse primer: 5′-GCTCAAAGCCTGCATCCCA-3′; site 3 (GCCTGAGGG, at −2548 bp) was amplified using forward primer 5′-ACACACACACACAGAGGCTA-3′ and reverse primer 5′- CCAATGCGGTTCAACAGACA-3′.

### Fluorochrome-conjugated Cholera Toxin Subunit B (CTB) injections

Three *AP-2δ+/+* and four *AP-2δ−/−* adult mice (3 month old matched males or matched females from the same litter) were used for the experiments. Animals were anesthetized using a mixture of ketamine and xylazine. Eyes were injected as follows: 2.5 μl of a 1 % emulsified solution of Alexa 488-conjugated cholera toxin subunit B (CTB, Molecular Probes, Life Technologies) into the vitreous chamber of the right eye and 2.5 μl of a 1 % emulsified solution of Alexa 594-conjugated CTB into the vitreous chamber of the left eye. Seven days post-injection, animals were euthanized with an overdose of pentobarbital sodium (euthanyl, Bimeda-MTC) and perfused transcardially with 4 % paraformaldehyde. The brains and eyes were dissected, fixed overnight in 4 % paraformaldehyde. Flattened whole-mounts were prepared by removing the retinas as intact cups, making four radial cuts to flatten the retinas, and mounting the retinas onto glass slides. Brains were cryoprotected in graded sucrose solutions (10 %, 20 %, 30%), then serially sectioned in the sagittal plane (30 μm sections), with 120–150 sections generated for each brain.

Each brain section from each of the seven CTB-injected mice was photographed using the Tile-scan function of the LSM program and a 10× objective (frame size of each tile = 512 × 512 pixels). This program generates a montage of all the scans taken for each brain section. The acquired images were used to analyze the extension of retinal projections into five main visual processing centers of the brain: suprachiasmatic nucleus (SCN), dorsal lateral geniculate (DLG) nucleus, ventral lateral geniculate nucleus - magnocellular part (VLG-mc), medial terminal nucleus (MTN) and superior colliculus (SC). The termination fields of the retinal projections were measured in two different ways: (i) “sum all”, V = Σ [S] (S = surface of the projections in individual sections) x thickness of each section. “sum all” was used when all sections were available for a particular structure, or (ii) “Cavalieri formula”, V = Σ [S] x d (with [S] = surface of the projections in the sections selected for calculation, and d = distance between the selected sections. The Cavalieri formula was used when some sections in the structure were unanalyzable.

The surface occupied by the termination fields of the retinal projections was calculated by first splitting the green and red channels. Each image was thresholded to increase signal (signal-to-background ratio of ~5:1) and to remove weakly-labeled isolated fibers. The remaining highly contrasted terminal fields of retinal projections in each image were precisely outlined and the surface of the selected area measured using the “Analyze-Measure-Area” subroutine of the ImageJ program (https://imagej.nih.gov/ij/). Repeated measurements of these selected areas yielded a variation of ≤5 %. Terminal field areas and volumes calculated after image processing did not differ significantly from those obtained with raw RGB images.

The lateral extent of each structure occupied by contralateral and ipsilateral projections was assessed by counting the total number of sections containing labeled axons from each projection. The anteroposterior (A-P) length of the projection field was obtained by drawing a straight line from the rostral to the caudal pole of the longest contralateral projection. The thickness of the contralateral projection to the SC refers to the largest of all values obtained in each section at a location ~750 μm caudal to the rostral pole of the SC. All values are given for the green projections (the projections issued from the eye that received CTB-Alexa 488) rather than the red projections because the green label was more vivid than the red label and was thus easier to measure, especially for sparse ipsilateral projections. Measuring contralateral and ipsilateral projections of the same color also eliminates possible disparities between red and green ocular injections. No significant difference was found between calculation modes.

### Electrophysiology recordings and analyses

Eight *AP-2δ+/+* and ten AP*-2δ−/−* adult mice (3–4 month old matched male and female littermates) were analyzed. Following 1 h dark-adaptation, animals were prepared for ERG recordings under dim red light. Mice were anesthetized with ketamine [75 mg/kg intraperitoneal (i.p.)] and xylazine (15 mg/kg i.p.), and pupils dilated with a topical solution of 1 % tropicamide. The active electrode (gold loop) was placed on the cornea while the reference electrode (platinum 30G needle) was inserted subdermally in the temporal ridge; the ground electrode (also a platinum 30G needle) was subdermally inserted at the level of the lower neck. First, dark-adapted responses were recorded by stimulating the retina with 19 stepwise pulses of increasing light intensity ranging from −5.22 to 2.86 log cds/m^2^ (logarithm of scotopic candela seconds/meter square). Ten minutes following transition from dark to light-adaptation (30 cd/m^2^ white light background), photopic responses were recorded by stimulating the retina with 11 stepwise pulses of increasing light intensity ranging from −1.63 to 2.86 log cds/m^2^, followed by flicker responses (stimuli of 1 log cds/m^2^ luminance presented at 11 stepwise increasing frequencies ranging from 3 to 60 Hz). Light adaptation was used to saturate rod photoreceptors and therefore isolate pure cone-driven responses. While ERGs were recorded bilaterally, a single eye per mouse was included in the analyses, which corresponded to the one yielding the highest mixed a-wave amplitude.

Light-adapted VER was recorded by placing the active electrode subdermally over the visual cortex located in the back of the skull. The reference electrode was placed between the 2 eyes with the ground electrode located at the base of the tail. The stimulus consisted of a full field flash of 1.32 log cds/m^2^ presented 200 times to produce an averaged trace with optimal signal to noise ratio.

### Statistical analysis

All values are given as mean ± SD. Differences in number of Brn3a + ve cells in the ganglion cell layer as well as differences in the projection areas were tested using the non-parametric Mann–Whitney U-test for independent samples. This test is used for analysis of small sample sizes and requires large differences (≥20 %) in order to show significance. For ERG data, statistical significance between the two groups was assessed using repeated-measures ANOVA with the Greenhouse-Geisser correction for sphericity. In cases when statistical differences were found for the whole data series, post hoc analyses were done between the two groups at individual stimulus strengths (for intensity response series) or frequency (for flicker) using the Bonferroni technique for multiple comparisons. GraphPad Prism (GraphPad Software, Inc., La Jolla, CA, USA) was used for calculations. *p* values are given for two-tailed, non-directional tests. Significance was set at *p* < 0.05.

## Abbreviations

AP-2, activating protein-2; ChIP, chromatin immunoprecipitation; CNS, central nervous system; CTB, cholera toxin subunit B; ERG, electroretinography; LGN, lateral geniculate nuclei; MTN, medial terminal nuclei; RGC, retinal ganglion cell; RHT, retino-hypothalamic tract; SC, superior colliculus; SCN, suprachiasmatic nuclei; VER, visually evoked response.

## Additional file

Additional file 1: Figure S1. Loss of Brn3c immunostaining in *AP-2*δ*−/−* retinal ganglion cells. Retinal tissue sections from *AP-2*δ*+/+* and *AP-2*δ*−/−* mice at E16.5, P1, P14 and adult were co-immunostained with anti-AP-2δ and anti-Brn3c (QQ8) antibodies followed by secondary antibodies conjugated to Alexa 488 and Alexa 555, respectively. Tissue sections were counterstained with DAPI Cells immunostained with the anti-Brn3c (QQ8) antibody in *AP-2*δ*−/−* retina are indicated by arrows. **Figure S2.** Loss of Brn3c expression in *AP-2*δ*−/−* P1 retina. Paraffin-embedded tissue sections from P1 *AP-2*δ*+/+* and *AP-2*δ*−/−* retina were immunostained with two anti-Brn3c antibody: mouse anti-Brn3c (Santa Cruz QQ8) and rabbit anti-Brn3c antibody (Sigma Atlas). The signal was detected using the Dako-Cytomation EnVision + anti-rabbit or anti-mouse secondary systems. Photographs were taken using a 20× lens and images merged. **Figure S3.** Brn3a labeling in P1 and P14 *AP-2*δ*+/+* and *AP-2*δ*−/−* retina. Retinal tissue sections were immunostained with anti-Brn3a antibody followed by secondary antibody conjugated to Alexa 555. Tissue sections were counterstained with DAPI. Photographs were taken with a 40× lens. **Figure S4.** Brn3a labeling in P21 and adult *AP-2*δ*+/+* and *AP-2*δ*−/−* retina. Retinal tissue sections were processed as described in Figure S3. **Figure S5.** Flat-mount of cholera toxin B (CTB)-injected retina. Retinas from *AP-2*δ*+/+* and *AP-2*δ*−/−* mice injected with CTB were flat-mounted onto glass slides. The dorsal central regions of the retinas are shown in the upper panels and the optic discs are shown in the lower panels. Photographs were taken with a 20× lens. Scale bar = 100 μm. **Figure S6.** AP-2δ and AP-2β immunostaining of P1 mouse brain. Sagittal tissue sections close to the midline of wild-type P1 mouse brain were immunostained with anti-AP-2δ and anti-AP-2β antibodies. The emerging stratum zonale (SZ), stratum griseum superficiale (SGS), stratum opticum or optic layer, intermediate and deep layers of the superior colliculus (SC) are indicated. The white layer of the SC separates the SC from 2 the periaqueductal gray (PAG). The insets on the left are magnified in the diagram on the right. Both AP-2β and AP-2δ are widely expressed in the SC. Scale bars = 100 μm. **Table S1.** Volume of contralateral projections to the MTN using raw RGB image data and thresholded image data. Abbreviations for additional files: NBL, neuroblastic layer; INBL, inner neuroblastic layer; INL, inner nuclear layer; GCL, ganglion cell layer; ONL, outer nuclear layer. (PDF 1923 kb)
