# Supplementary material for: Loss of AP-2delta reduces retinal ganglion cell numbers and axonal projections to the superior colliculus
Source: Mol Brain. 2016 Jun 4;9:62. doi: 10.1186/s13041-016-0244-0 (PMC4893287; doi:10.1186/s13041-016-0244-0)
Supplement: Additional file 1: Figure S1. — Loss of Brn3c immunostaining in AP-2δ−/− retinal ganglion cells. Retinal tissue sections from AP-2δ+/+ and AP-2δ−/− mice at E16.5, P1, P14 and adult were co-immunostained with anti-AP-2δ and anti-Brn3c (QQ8) antibodies followed by secondary antibodies conjugated to Alexa 488 and Alexa 555, respectively. Tissue sections were counterstained with DAPI Cells immunostained with the anti-Brn3c (QQ8) antibody in AP-2δ−/− retina are indicated by arrows. Figure S2. Loss of Brn3c expression in AP-2δ−/− P1 retina. Paraffin-embedded tissue sections from P1 AP-2δ+/+ and AP-2δ−/− retina were immunostained with two anti-Brn3c antibody: mouse anti-Brn3c (Santa Cruz QQ8) and rabbit anti-Brn3c antibody (Sigma Atlas). The signal was detected using the Dako-Cytomation EnVision + anti-rabbit or anti-mouse secondary systems. Photographs were taken using a 20× lens and images merged. Figure S3. Brn3a labeling in P1 and P14 AP-2δ+/+ and AP-2δ−/− retina. Retinal tissue sections were immunostained with anti-Brn3a antibody followed by secondary antibody conjugated to Alexa 555. Tissue sections were counterstained with DAPI. Photographs were taken with a 40× lens. Figure S4. Brn3a labeling in P21 and adult AP-2δ+/+ and AP-2δ−/− retina. Retinal tissue sections were processed as described in Figure S3. Figure S5. Flat-mount of cholera toxin B (CTB)-injected retina. Retinas from AP-2δ+/+ and AP-2δ−/− mice injected with CTB were flat-mounted onto glass slides. The dorsal central regions of the retinas are shown in the upper panels and the optic discs are shown in the lower panels. Photographs were taken with a 20× lens. Scale bar = 100 μm. Figure S6. AP-2δ and AP-2β immunostaining of P1 mouse brain. Sagittal tissue sections close to the midline of wild-type P1 mouse brain were immunostained with anti-AP-2δ and anti-AP-2β antibodies. The emerging stratum zonale (SZ), stratum griseum superficiale (SGS), stratum opticum or optic layer, intermediate and deep layers of the superior colliculus (SC) are [file 13041_2016_244_MOESM1_ESM.pdf]

Supplementary figure legends

Figure S1. Loss of Brn3c immunostaining in *AP-2δ-/-* retinal ganglion cells. Retinal tissue sections from *AP-2δ+/+* and *AP-2δ-/-* mice at E16.5, P1, P14 and adult were co-immunostained with anti-AP-2δ and anti-Brn3c (QQ8) antibodies followed by secondary antibodies conjugated to Alexa 488 and Alexa 555, respectively. Tissue sections were counterstained with DAPI to label the nuclei. Photographs were taken with a Zeiss LSM710 confocal microscope. Cells immunostained with the anti-Brn3c (QQ8) antibody in *AP-2δ-/-* retina are indicated by arrows. Abbreviations: NBL, neuroblastic layer; INBL, inner neuroblastic layer; INL, inner nuclear layer; GCL, ganglion cell layer; ONL, outer nuclear layer.

Figure S2. Loss of Brn3c expression in *AP-2δ-/-* P1 retina. Paraffin-embedded tissue sections from P1 *AP-2δ+/+* and *AP-2δ-/-* retina were immunostained with two anti-Brn3c antibody: mouse anti-Brn3c (Santa Cruz QQ8) and rabbit anti-Brn3c antibody (Sigma Atlas). The signal was detected using the Dako-Cytomation EnVision+ anti-rabbit or anti-mouse secondary systems. Tissues were counterstained with the nuclear stain hematoxylin. Photographs were taken using a 20X lens. Images of the entire retina sections were assembled from multiple overlapping photographs using the Adobe Photoshop merge function.

Figure S3. **Brn3a labeling in *AP-2δ+/+* and *AP-2δ-/-* retina.** Retinal tissue sections from *AP-2δ+/+* and *AP-2δ-/-* mice at P1 and P14 were immunostained with anti-Brn3a antibody followed by secondary antibody conjugated to Alexa 555. Tissue sections were

counterstained with DAPI to label the nuclei. Photographs were taken with a Zeiss LSM710 confocal microscope equipped with a 40X lens. Abbreviations: INBL, inner neuroblastic layer; INL, inner nuclear layer; GCL, ganglion cell layer; ONL, outer nuclear layer.

Figure S4. **Brn3a labeling in *AP-2δ+/+* and *AP-2δ-/-* retina.** Retinal tissue sections from *AP-2δ+/+* and *AP-2δ-/-* mice at P21 and adult were immunostained with anti-Brn3a antibody followed by secondary antibody conjugated to Alexa 555. Tissue sections were counterstained with DAPI to label the nuclei. Photographs were taken with a Zeiss LSM710 confocal microscope equipped with a 40X lens. Abbreviations: INL, inner nuclear layer; GCL, ganglion cell layer; ONL, outer nuclear layer.

Figure S5. Flat-mount of cholera toxin B (CTB)-injected retina. Retinas from *AP-2δ+/+* and *AP-2δ-/-* mice injected with CTB were flat-mounted onto glass slides. The dorsal central regions of the retinas are shown in the upper panels and the optic discs are shown in the lower panels. Photographs were taken with a Zeiss LSM710 confocal microscope equipped with 20X lens. Scale bar = 100 μm.

Figure S6. AP-2δ and AP-2β immunostaining of P1 mouse brain. Sagittal tissue sections close to the midline of wild-type P1 mouse brain were immunostained with anti-AP-2δ and anti-AP-2β antibodies. The emerging stratum zonale (SZ), stratum griseum superficiale (SGS), stratum opticum or optic layer, intermediate and deep layers of the superior colliculus (SC) are indicated. The white layer of the SC separates the SC from

the periaqueductal gray (PAG). The insets on the left are magnified in the diagram on the right. The inferior colliculus (IC) is strongly positive for AP-2δ, but not for AP-2β. Both AP-2β and AP-2δ are widely expressed in the SC. Scale bars = 100 μm.

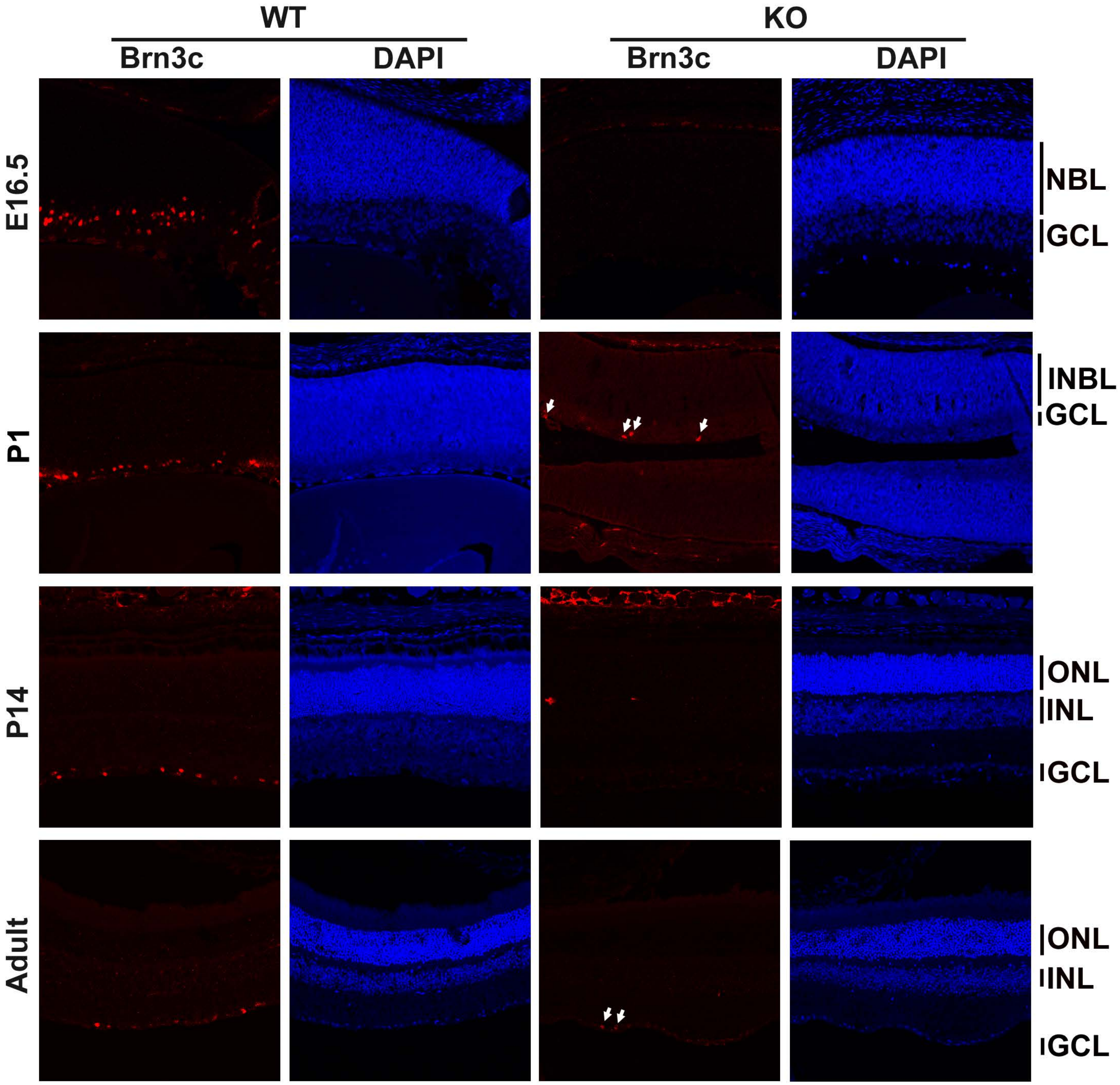

Supplementary Figure S1

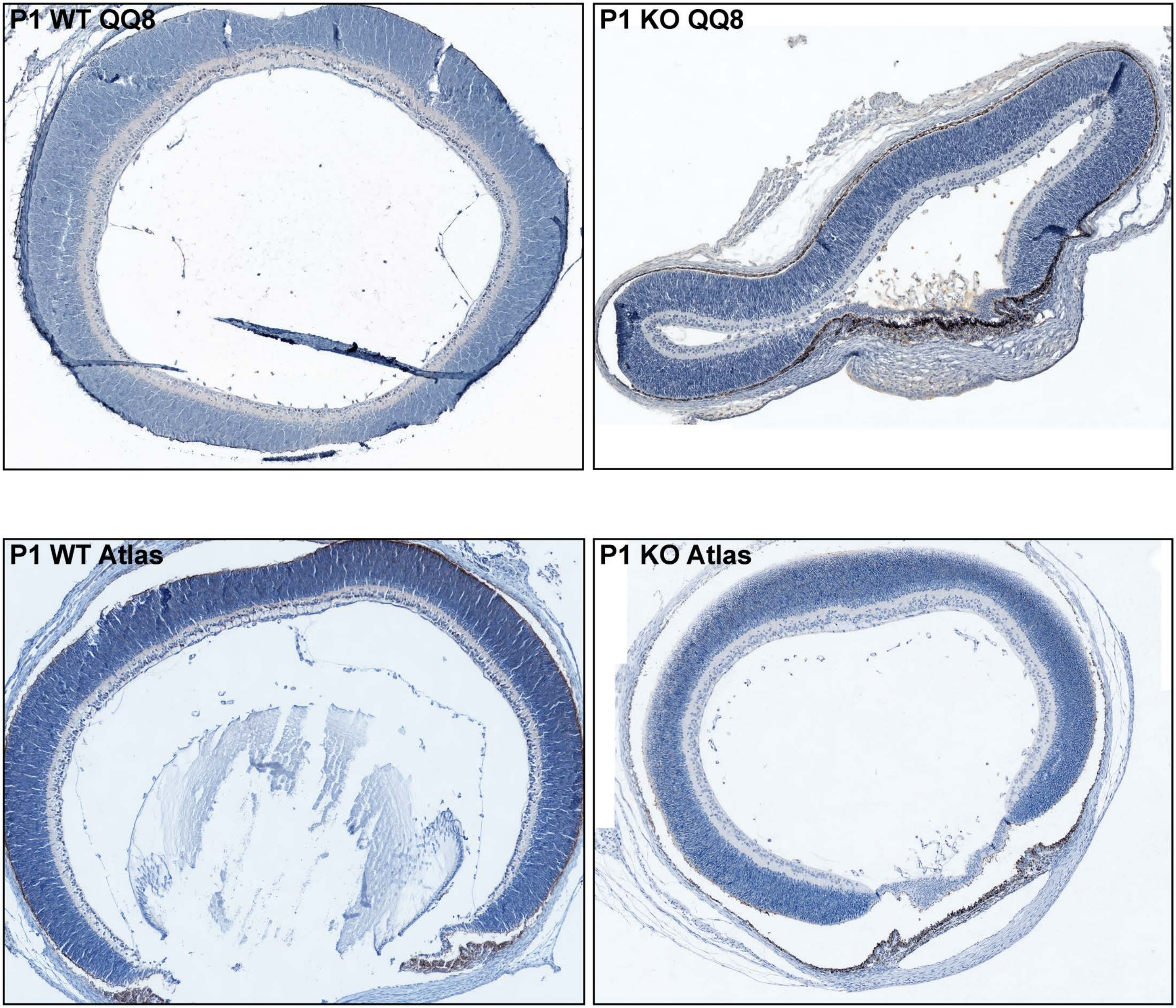

Supplementary Figure S2

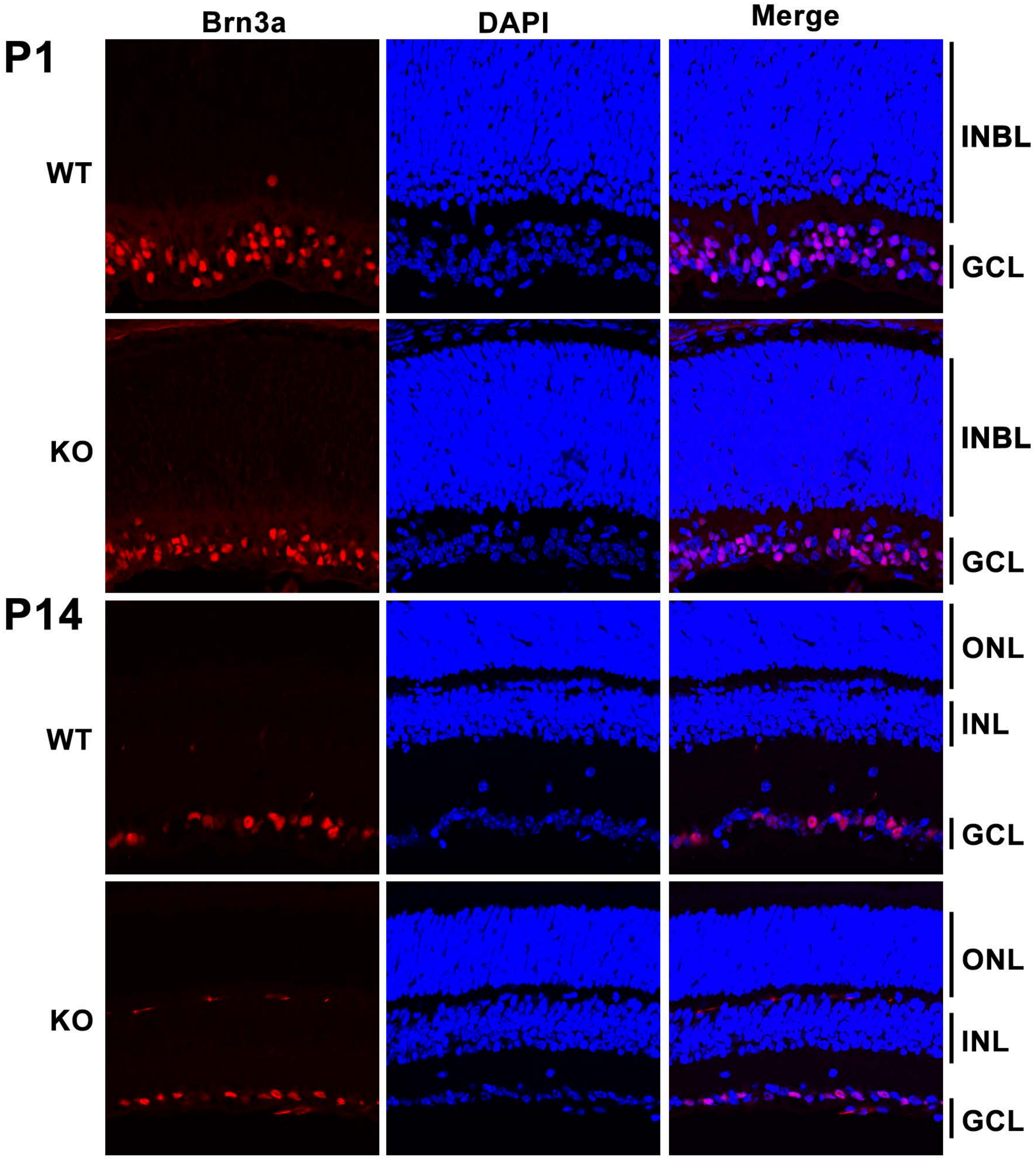

Supplementary Figure S3

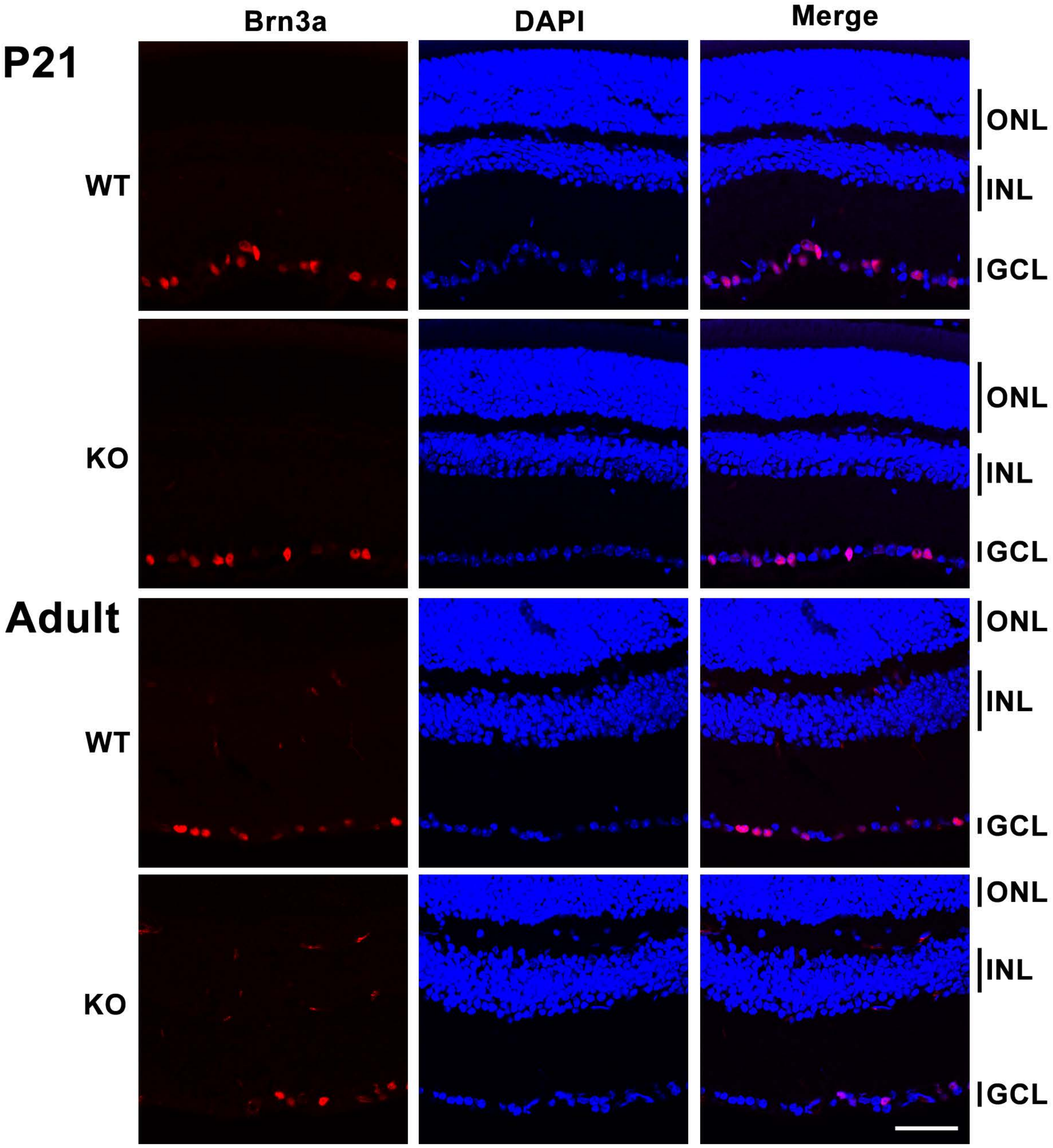

Supplementary Figure S4

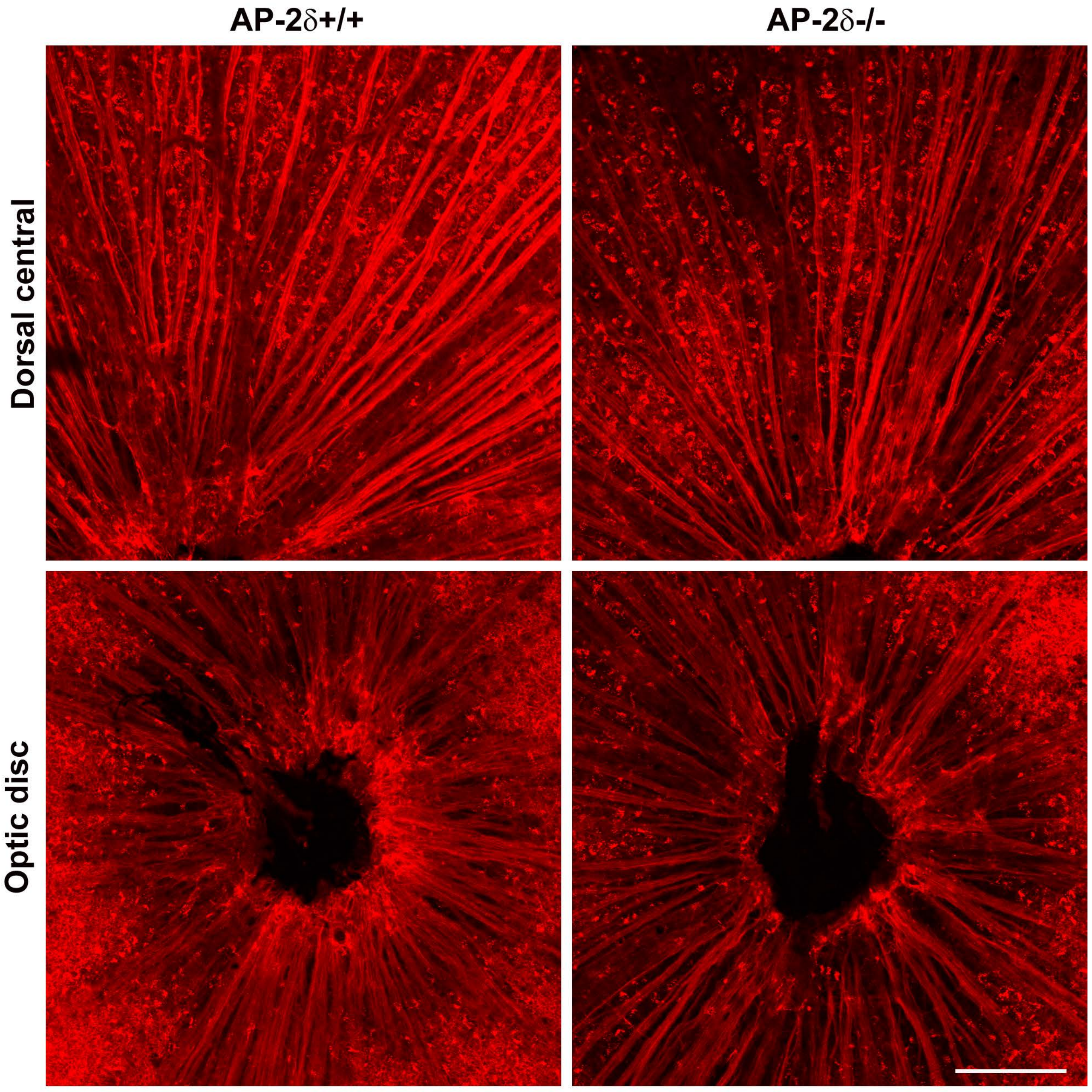

Supplementary Figure S5

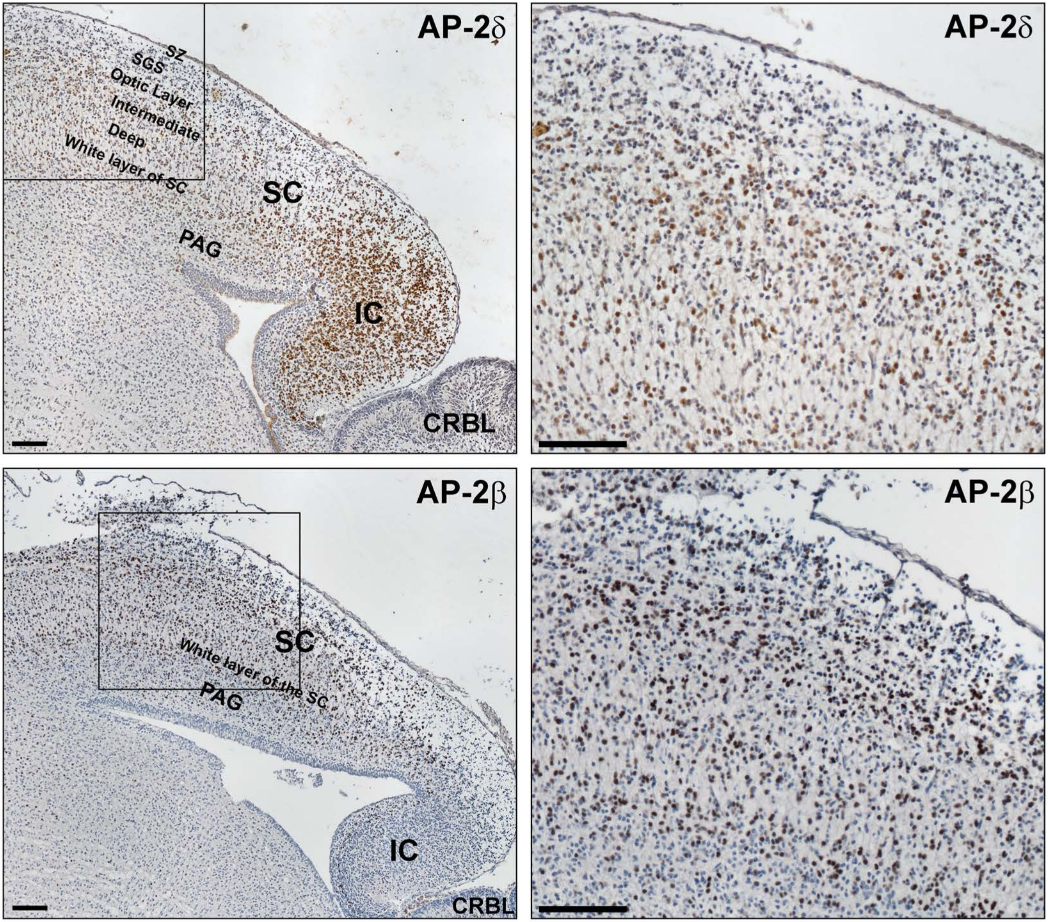

Supplementary Figure S6

Table S1. Volume of contralateral projections to the MTN using raw RGB image data and thresholded image data.

Animal	Raw RGB image (mm^3)	Thresholded image (from Table 1) (mm^3)
W4-WT	0.00920	0.00912
AE1-WT	0.00726	0.00755
AF2-WT	0.00753	0.00755
T1-KO	0.0117	0.0112
W1-KO	0.0112	0.0105
AE2-KO	0.0106	0.0096
AF1-KO	0.00905	0.00915
